# A Tumor Vascular‐Targeted Interlocking Trimodal Nanosystem That Induces and Exploits Hypoxia

**DOI:** 10.1002/advs.201800034

**Published:** 2018-05-28

**Authors:** Xin Luan, Ying‐Yun Guan, Hai‐Jun Liu, Qin Lu, Mei Zhao, Duxin Sun, Jonathan F. Lovell, Peng Sun, Hong‐Zhuan Chen, Chao Fang

**Affiliations:** ^1^ Hongqiao International Institute of Medicine Shanghai Tongren Hospital and Department of Pharmacology and Chemical Biology Institute of Medical Sciences Shanghai Jiao Tong University School of Medicine (SJTU‐SM) 280 South Chongqing Road Shanghai 200025 China; ^2^ Department of Pharmaceutical Sciences College of Pharmacy University of Michigan Ann Arbor MI 48105 USA; ^3^ Institute of Interdisciplinary Integrative Biomedical Research Shanghai University of Traditional Chinese Medicine 1200 Cailun Road Shanghai 201210 China; ^4^ Department of Pharmacy Ruijin Hospital SJTU‐SM, 197 Rui Jin Er Road Shanghai 200025 China; ^5^ Department of Pharmacy Shanghai University of Medicine & Health Sciences 279 Zhouzhu Road Shanghai 201318 China; ^6^ Department of Biomedical Engineering University at Buffalo State University of New York Buffalo NY 14260 USA; ^7^ Department of General Surgery Shanghai Tongren Hospital SJTU‐SM, 1111 Xianxia Road Shanghai 200336 China

**Keywords:** graphene oxide, hypoxia‐activated prodrug, photodynamic therapy, trimodal therapy, tumor hypoxia

## Abstract

Vascular‐targeted photodynamic therapy (VTP) is a recently approved strategy for treating solid tumors. However, the exacerbated hypoxic stress makes tumor eradication challenging with such a single modality approach. Here, a new graphene oxide (GO)‐based nanosystem for rationally designed, interlocking trimodal cancer therapy that enables VTP using photosensitizer verteporfin (VP) (1) with codelivery of banoxantrone dihydrochloride (AQ4N) (2), a hypoxia‐activated prodrug (HAP), and HIF‐1α siRNA (siHIF‐1α) (3) is reported. The VTP‐induced aggravated hypoxia is highly favorable for AQ4N activation into AQ4 (a topoisomerase II inhibitor) for chemotherapy. However, the hypoxia‐induced HIF‐1α acts as a “hidden brake,” through downregulating CYP450 (the dominant HAP‐activating reductases), to substantially hinder AQ4N activation. siHIF‐1α is rationally adopted to suppress the HIF‐1α expression upon hypoxia and further enhance AQ4N activation. This trimodal nanosystem significantly delays the growth of PC‐3 tumors in vivo compared to the control nanoparticles carrying VP, AQ4N, or siHIF‐1α alone or their pairwise combinations. This multimodal nanoparticle design presents, the first example exploiting VTP to actively induce hypoxia for enhanced HAP activation. It is also revealed that HAP activation is still insufficient under hypoxia due to the hidden downregulation of the HAP‐activating reductases (CYP450), and this can be well overcome by GO nanoparticle‐mediated siHIF‐1α intervention.

## 1 Introduction

The development of antiangiogenic agents (AAs) has yielded significant clinical results in improved progression‐free survival and overall survival. Ten new drugs (seven small kinase inhibitors, two antibodies, and one fusion protein) are approved by FDA for multiple cancer indicators.^1^ Photodynamic therapy (PDT) is a clinically approved, minimally invasive, light‐triggered cancer therapeutic method, and the antivascular effects of PDT are known to contribute greatly to its efficacy.^2^ Efforts have been made to combat cancers particularly with vascular‐targeted PDT (VTP). Typically, VTP is performed by irradiating the target tissue a short duration after photosensitizer (PS) administration (e.g., 15 min after intravenous injection of PS). In this manner, the PS is passively distributed mainly in the vascular compartment.^3^ The representative product, WST‐11 (TOOKAD soluble, padeliporfin) developed by STEBA Biotech, has entered into phase III in Europe and recently been approved for use in early‐stage prostate cancer in Mexico.^4^ In order to strengthen the vascular‐targeted efficacy, other attempts have involved conjugating vascular targeting ligands to the PS or PS nanocarrier to directly target the PS to tumor neovasculature.^5^ The vascular‐targeting ligands like tripeptide Arg‐Gly‐Asp (RGD) have been widely used to target nanoparticles to tumor vessels.^6^

However, tumor hypoxic stress will be inevitably exacerbated by the antivascular effects and oxygen consumption in the tumor microenvironment (TME) after PDT. This usually leads to compromised efficacy and clinical performance of PDT[[qv: 3a,7]] and other antiangiogenic strategies.^8^ One emerging modality to overcome hypoxia and the associated stress response is to combine PDT with chemotherapy (chemophototherapy),^9^ among which the combinations of PDT and AA have received more attention.^10^ However, some preclinical attempts still yielded unsatisfactory outcome: 1) Compared to PDT alone, PDT followed with bevacizumab or sunitinib (two FDA‐approved AAs) did not further impede tumor growth.^11^ We speculate that the antivascular effects of PDT seriously prevented the tumor perfusion of the follow‐up AAs. 2) While if the administration sequence was reversed, precedently dosed bevacizumab even antagonized the effects of the follow‐up PDT.^11^ This may be caused by the antiangiogenic effects of bevacizumab, which could either directly compromise the PS perfusion or aggravate hypoxia and thus extremely impair the potency of the oxygen‐dependent PDT.

Here, we developed a new VTP‐based strategy in which the VTP‐induced hypoxia was smoothly exploited for enhanced cancer treatment. This was achieved by an engineered cyclo (Arg‐Gly‐Asp‐D‐Phe‐Lys) (c(RGDfK)) peptides modified graphene oxide (GO)‐based nanosystem, codelivering verteporfin (VP), banoxantrone dihydrochloride (AQ4N), and HIF‐1α siRNA (siHIF‐1α) for trimodal combination therapy:

(1) VP is a photosensitizer with a long absorbance at 690 nm. VP‐mediated PDT has been approved for age‐related macular degeneration,^2^ and also investigated against multiple cancers.^12^
(2) AQ4N is a representative hypoxia‐activated prodrug (HAP). Besides hypoxia, the activation of AQ4N also depends on cytochrome P450 (CYP450) activating reductases, which are also dominantly responsible for the activation of most other HAPs.^13^ Two main CYP450 enzymes, CYP1A1 and 2B6, are shown to metabolize AQ4N into AQ4 (a potent inhibitor of topoisomerase II) efficiently (Figure S1, Supporting Information).^14^ However, O_2_ blocks this activation process by outcompeting AQ4N for heme‐centered active site of CYP450, conferring the selectivity of AQ4N in eradicating hypoxic tumor cells.^15^

Unfortunately, the development of multiple HAPs is experiencing bottleneck in clinical trials.[[qv: 13b]] One major hindrance is the low levels of hypoxia in heterogeneous tumor microenvironment,[[qv: 13b,16]] which leads to low efficient activation of HAPs. In this study, we aimed to use VP‐mediated VTP to actively induce aggravated hypoxia in tumor sites and enhance AQ4N activation.

(3) Elevated HIF‐1α expression is one of the most important events under tumor hypoxic stress, which mediates various adaptive responses for tumor to survive under hypoxia.[[qv: 13a]] Noticeably, HIF‐1α can also downregulate CYP450 via competitive binding with HIF‐1β against aryl hydrocarbon receptor (AhR).^17^ In this manner, HIF‐1α actually acts as a “hidden brake” to substantially hinder AQ4N activation under hypoxia. However, this issue has not ever been considered in the previous report involving nanoparticle‐mediated combination therapy of PDT and AQ4N.^18^ We here adopted siHIF‐1α to suppress HIF‐1α, therefore upregulate CYP450, and enhance the AQ4N activation under hypoxia.

We chose GO as the nanoscaffold, as it is well biocompatible and has been widely used as multifunctional nanocarrier for drug and gene delivery.^19^ VP and AQ4N were closely absorbed on GO through π–π stacking and hydrophobic interactions. siHIF‐1α was efficiently condensed on the polyethylenimine (PEI) which was linked to GO by *N*‐(3‐Dimethylaminopropyl‐*N*'‐ethylcarbodiimide) hydrochloride/*N*‐hydroxysulfosuccinimide sodium salt chemistry. c(RGDfK) peptides are classical high avidity (Kd ≈ 40 × 10^−9^ m) ligands to target α_v_β_3_,^20^ overexpressed on the surface of tumor vascular endothelial cells (ECs) and many tumor cells such as PC‐3 prostate cancer cells.^21^ The RGD modification‐mediated nanoparticle uptake in α_v_β_3_ expressed cells has been widely proved in previous reports.^22^ c(RGDGfK)‐capped eight‐arm polyethylene glycol (PEG) used here conferred both the stealth property and tumor vessel and tumor cell dual‐targeted merits of the nanosystem.

**Figure 1:**
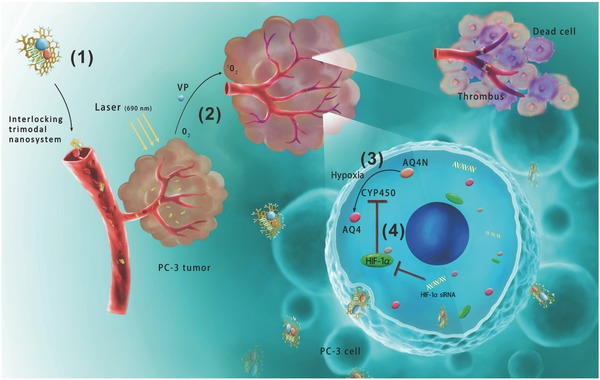
Schematic illustration of the working model of the interlocking trimodal graphene‐oxide‐based nanosystem. 1) The trimodal nanosystem, c(RGDfK)‐ppGO/VP‐AQ4N‐siHIF‐1α, targets to the PC‐3 tumors after being intravenously injected into the mice. 2) VP‐based VTP with 690 nm irradiation results in tumor vessel occlusion and aggravated hypoxia, 3) which effectively activates AQ4N into cytotoxic AQ4. 4) However, the increased HIF‐1α upon VTP downregulates CYP450 activating reductases, acting as a “hidden brake” to prevent AQ4N activation under hypoxia. Through knocking down HIF‐1α, the codelivered siHIF‐1α can upregulate CYP450 expression to further strengthen AQ4N activation.

We hypothesize that (1) VP‐mediated VTP can lead to aggravated hypoxia through antivascular effects, which can be smartly used for highly effective AQ4N activation into AQ4 for enhanced chemotherapy; and (2) siHIF‐1α can suppress HIF‐1α expression upon VTP‐induced acute hypoxia, and furthermore increase the levels of CYP1A1 and 2B6 essential for AQ4N activation. Our strategy is proved to be effective in a prostate cancer model. The working model of the mechanism‐based, interlocking trimodal GO‐based nanosystem is schematically illustrated in **Figure** **1**.

Previous nanoparticle‐based research mainly focused on relieving tumor hypoxia to enhance chemotherapy, radiotherapy, or PDT.^23^ Typically, using calatase to produce endogenous H_2_O_2_‐derived O_2_ 24 or perfluorocarbon to delivery exogenous O_2_ 25 are two main methods. Several efforts were also made to utilize hypoxia,^23^ such as HAPs therapy^18^, ^26^ or hypoxia‐triggered drug delivery.^27^ However, to the best of our knowledge, this is the first effort exploiting VTP (a clinically relevant strategy) to induce hypoxia for successfully enhanced HAP activation. This smart design for the combination of VTP and HAP holds promise for clinical translation and meanwhile overcoming their respective limitation of single modality approach. Moreover, the important ‘hidden brake’ role of increased HIF‐1α upon hypoxia in hindering HAP activation by downregulating CYP450 is first revealed, and this issue is well solved in this study through using the GO nanoparticle‐mediated siHIF‐1α intervention.

## 2 Results

### 2.1 Nanoparticle Characterization and Cellular Uptake

**Figure 2:**
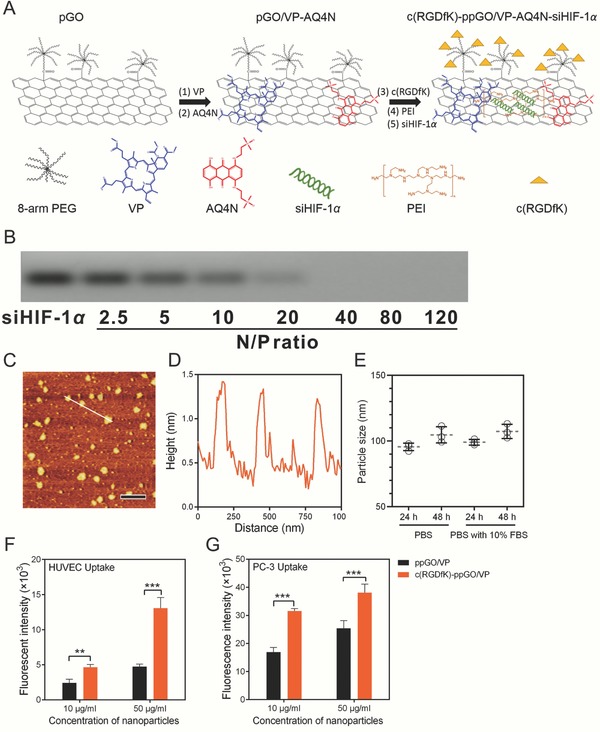
Preparation and characterization of the trimodal nanosystem. A) Schematic illustration of the preparation process. Note: pGO, PEG‐GO; ppGO, pGO conjugated with PEI. B) Electrophoretic mobility shift assay of the siHIF‐1α complexed with c(RGDfK)‐ppGO/VP‐AQ4N at different N/P ratios. C) AFM photograph (bar, 500 nm). D) The height profile of the AFM image showed that the nanosystem was 1–2 layered sheets. E) Colloid stability of the nanosystem in PBS and PBS with 10% FBS after 24 and 48 h incubation at 37 °C. The data are presented as means ± s.d. of three independent replicates. The nanoparticle uptake in F) HUVECs and G) PC‐3 cells after 2 h incubation at 37 °C was observed and assayed by quantifying the intracellular fluorescence intensity on the Thermo Scientific ArrayScan XTI High Content Analysis Reader. The VP (Ex: 650 nm, Em: 690 nm) was used as the fluorescent probe. The mean ± s.d. from four independent replicates is shown. **p < 0.01, ***p < 0.001.

The trimodal nanosystem was stepwise engineered as illustrated in **Figure** **2**A. The three active molecules (VP, AQ4N, and siHIF‐1α) were coloaded onto c(RGDfK) modified nano‐GO, forming a targeted combination nanosystem, namely c(RGDfK)‐ppGO/VP‐AQ4N‐siHIF‐1α. The loading of VP and AQ4N on the nanoparticles was identified and quantified with UV–vis–NIR absorbance detection, and the absorbance peaks were at 410 nm for VP and 610 nm for AQ4N (Figure S2, Supporting Information). The drug loading (DL%) was 7% for VP and 35% for AQ4N. The electrophoretic mobility shift assay indicated that the complete adsorption of siHIF‐1α on the c(RGDfK)‐ppGO/VP‐AQ4N was obtained when the N/P ratio was greater than 40 (Figure 2B). Thus, N/P of 40 was selected in the following study involved with nanoparticles containing siRNA. Atomic force microscopy (AFM) images showed that the nanosystem had a sheet structure (Figure 2C), and the thickness 1–2 nm indicated a structure characteristic of a single or two layered sheets (Figure 2D), according to previous work.^28^ This nanosystem had hydrodynamic size of 91.3 nm and zeta potential of 16.2 mV (Table S1, Supporting Information).

The nanosystem had good colloidal stability in phosphate buffered saline (PBS; pH 7.4, 0.01 m) and PBS containing 10% fetal bovine serum (FBS) (Figure 2E). Also, it slowly released VP and AQ4N with relatively low burst release, implying the less drug leakage before the nanoparticles target to the tumor in vivo (Figure S3, Supporting Information). Other nanoparticles carrying only one molecule or their pairwise combinations, as well as the empty nanocarrier, were also prepared when the corresponding molecules were included during the preparation. Their sizes and zeta potentials were also measured using a Zetasizer Nano ZS instrument (Table S1, Supporting Information). The contents of PEI and PEG in the nanocarrier (ppGO) were also quantified. PEI was determined to be –36% (w/w) by assaying the cuprammonium complex formed by PEI and copper (II) ions at 285 nm (Figure S4, Supporting Information).^29^ The PEG content was determined to be –15% (w/w), using the PEGylated protein ELISA kit (Enzo Life Sciences, Farmingdale, NY, USA).^30^ The c(RGDfK) content in the targeted nanocarrier (c(RGDfK)‐ppGO) was estimated to be –3.4% (w/w) through determining the conjugated peptides using the CBQCA Protein Quantitation Kit (Thermo Fisher Scientific, Shanghai, China).^31^

Efficient nanoparticle uptake in the targeted cells is important for VP, AQ4N, and siHIF‐1α to exert their activity, as all their targets locate inside the cells. We then evaluated the uptake of the nanocarrier, using the VP as the fluorescent probe, in human umbilical vein endothelial cells (HUVECs) or PC‐3 cells, both of which highly express integrin α_v_β_3_.^21^, ^32^ As expected, c(RGDfK) peptides improved the nanoparticle uptake in HUVECs and PC‐3 cells at both low (10 µg mL^−1^) and high (50 µg mL^−1^) nanoparticle concentrations, conferring the dual‐targeting property (Figure 2F,G).

### 2.2 Targeted Photodynamic Toxicity to Both HUVECs and PC‐3 Cells

**Figure 3:**
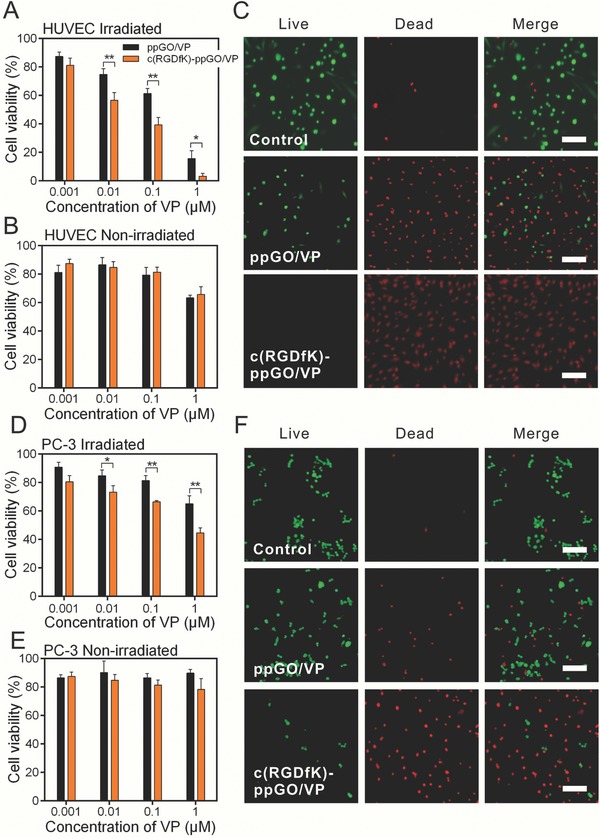
The viabilities of HUVECs and PC‐3 cells after photodynamic treatment with c(RGDfK)‐ppGO/VP or ppGO/VP. The viabilities of A,B) HUVECs and D,E) PC‐3 cells after treated with the nanoparticles and light irradiation (690 nm, 30 mW cm^−2^, 10 min) or not were evaluated using CCK‐8 assay. C) HUVECs and F) PC‐3 cells after PDT treatment with the nanoparticles containing 1 × 10^−6^ m VP were stained with LIVE/DEAD cell viability/cytotoxicity kit. The representative photographs of the cells were shown. Bar, 25 µm. The mean ± s.d. from three independent replicates is shown. *p < 0.05, **p < 0.01.

Next, we evaluated if the targeted cellular uptake can contribute significant VP‐mediated phototoxicity to both HUVECs and PC‐3 cells. The production of singlet oxygen was detected using danthracene‐9,10‐diyl‐bis‐methylmalonate (ADMA) to verify the PDT performance (Figure S5, Supporting Information).^33^ It showed photodynamic treatment with c(RGDfK)‐ppGO/VP (690 nm, 30 mW cm^−2^, 10 min) resulted in much higher toxicity to both HUVECs and PC‐3 cells compared to nontargeted ppGO/VP at VP concentrations of 0.01–1 × 10^−6^ m (**Figure** **3**A,D). These results were in agreement with the observation of calcein‐AM and PI dual staining assay (Figure 3C,F). Noticeably, HUVECs were much more sensitive to PDT treatment than PC‐3 cells. Around 50% PC‐3 cells remained viable after light irradiation with 1 × 10^−6^ m VP from c(RGDfK)‐ppGO/VP; however, almost all HUVECs lost their viabilities under the same condition. The superior damage to the endothelial cells is the requisite for VTP‐induced tumor vessel occlusion and blood stasis, and the injury to the PC‐3 tumor cells may partially benefit for inhibiting the tumor growth. Both c(RGDfK)‐ppGO/VP and ppGO/VP without 690 nm laser irradiation caused much less toxicity to the two targeted cells, implying that ppGO is well biocompatible, and the VP‐mediated phototoxicity dominantly contributed the cytotoxicity (Figure 3B,E).

### 2.3 Lysosomal Escape, Protein Expression of HIF‐1α and CYP450, and AQ4N Cytotoxicity in PC‐3 Cells

**Figure 4:**
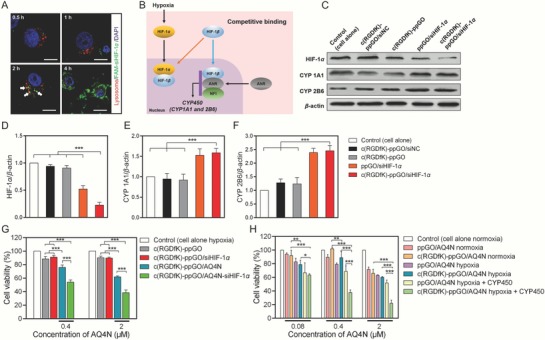
Lysosomal escape, protein expression of HIF‐1α and CYP450, and AQ4N cytotoxicity in PC‐3 cells. A) Intracellular trafficking of FAM‐labeled siHIF‐1α (green, Ex: 488 nm, Em: 520 nm) loaded in c(RGDfK)‐ppGO was observed using CLSM. The co‐localization (yellow dots indicated with white arrows) of the green dots and the red LysoTracker‐labeled lysosomes (Ex: 577 nm, Em: 590 nm) appeared at 2 h, showing the trapping of the siHIF‐1α‐condensed nanocarrier inside the lysosomes. The lyososomal escape was observed at 4 h after incubation. Note that at this time most of the lysosomes were swelled and ruptured, and could not be well labeled with LysoTracker. Bar, 10 µm. B) Schematic illustration of the mechanism how hypoxia negatively regulates CYP450. HIF‐1β, also known as AhR (aryl hydrocarbon receptor) nuclear translocator, can partner with AhR and NFI (nuclear factor 1) to form a heterotrimer and then binds to the promoter of CYP450 genes. However, hypoxia leads to high expression of HIF‐1α, which can translocate into the nucleus where it dimerizes with HIF‐1β, thus decreases the availability of HIF‐1β and causes a downregulation of CYP450. C) Western blot assay indicated that the expressions of HIF‐1α and CYP1A1 and 2B6 (two main AQ4N activating reductases) in hypoxic PC‐3 cells showed the opposite changing trend after siHIF‐1α treatment. siNC, negative control siRNA with a scrambled sequence. D–F) Statistical assay of the relative protein contents. The data are presented as means ± s.d. (*n* = 3), ***p < 0.001. G) siHIF‐1α (150 × 10^−9^ m) increased the AQ4N toxicity to hypoxic PC‐3 cells, although it alone was nontoxic at the tested dose. The data are presented as means ± s.d. (*n* = 3) ***p < 0.001. H) Exogenously added CYP450 enzymes from rat liver microsomes were beneficial for AQ4N‐mediated toxicity to hypoxic PC‐3 cells. The cell viabilities were detected using CCK‐8 assay. The data are presented as means ± s.d. (*n* = 3) *p < 0.05, **p < 0.01, ***p < 0.001.

Efficient escape from lysosomes into the cytosol is vital for nonviral gene delivery nanocarrier, which can prevent the siRNA from degradation by lysosomal nucleases.^34^ The trafficking of the c(RGDfK)‐ppGO/FAM‐siHIF‐1α in PC‐3 cells was investigated under confocal laser scanning microscopy (CLSM) (**Figure** **4**A). At 0.5 and 1 h, we can see fluorescent green dots (FAM‐siHIF‐1α condensed on the nanoparticles) appeared in the cells. At 2 h, the co‐localization (yellow) of the green dots and the red LysoTracker‐labeled lysosomes can be well observed, showing the trapping of the siHIF‐1α‐condensed nanocarrier inside the lysosomes. At 4 h, diffusive green regions instead of spotty fluorescence signaling spread inside the cytosol. In this scenario, most of the lysosomes were swelled and ruptured, and could not be well labeled with LysoTracker.^35^ Here, PEI offered the “proton sponge” effect for the successful escape of the nanoparticles from the lysosomes and the follow‐up efficient release of free FAM‐siHIF‐1α in the cytoplasm.

Then, we investigated if the delivered siHIF‐1α could suppress HIF‐1α expression under hypoxia and thereafter upregulate the contents of CYP1A1 and 2B6 (two main AQ4N activating enzymes),^14^ because HIF‐1α can take away the availability of HIF‐1β for AhR binding and thereafter reduce CYP450 expression (Figure 4B).^17^ We assayed the expression of HIF‐1α and CYP1A1 and 2B6 using Western blot (Figure 4C–F). It showed that siHIF‐1α significantly decreased the HIF‐1α expression in hypoxic PC‐3 cells, and this effect was more obvious as for the c(RGDfK)‐modified targeted nanosystem. Consistent with previous reports,^17^, ^36^ HIF‐1α inhibition led to increased expressions of CYP1A1 and 2B6, which can be favorable for AQ4N activation.[[qv: 13a]]

We then examined if siHIF‐1α could directly increase the AQ4N cytotoxicity. As expected, the involvement of siHIF‐1α significantly enhanced the AQ4N toxicity to hypoxic PC‐3 cells (Figure 4G). These results may be ascribed to the role of siHIF‐1α in upregulating the CYP450 enzymes in PC‐3 cells (Figure 4B–F). Noticeably, siHIF‐1α alone did not cause obvious cytotoxicity under the tested condition (Figure 4G).

We also explored the effects of the exogenously added CYP450 enzymes on AQ4N cytotoxicity (Figure 4H). It showed that under hypoxia, the AQ4N toxicity to PC‐3 cells was dramatically increased in the presence of CYP450 enzymes from rat liver microsomes, and this effect was more obvious in the cells treated with the c(RGDfK)‐modified targeted nanoparticles. Although hypoxia alone without the added CYP450 also increased the AQ4N cytotoxicity; however, this effect was relatively weak. These results proved that increased CYP450 enzymes are extremely beneficial for enhanced AQ4N activation and cytotoxicity under hypoxia, which are in good agreement with previous literature.^37^

### 2.4 Targeted Distribution of the c(RGDfK)‐Modified Nanosystem in PC‐3 Xenograft

**Figure 5:**
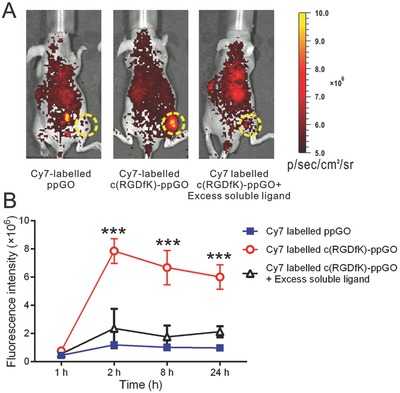
c(RGDfK) modification facilitated the nanoparticle accumulation in tumors. A) Male BALB/c nude mice bearing PC‐3 tumor (150 mm^3^) were given a single intravenous injection of Cy7‐labeled targeted or nontargeted nanoparticles (containing Cy7 0.4 mg kg^−1^). The mice in another group were coinjected with targeted nanoparticle and 50‐fold molar excess free c(RGDfK) peptides. Mice with in vivo Cy7 fluorescence were imaged at per‐determined time after injection using the Xenogen IVIS 200 system, and the representative images at 2 h were shown. The tumors were indicated in yellow circles. B) Statistical assay of the fluorescence intensity in the tumor regions at 1, 2, 8, and 24 h after injection. Data are presented as mean ± s.d. (*n* = 3) ***p < 0.001 compared to the other two groups.

Next, we investigated the in vivo tumor targeting feature of the c(RGDfK)‐conjugated nanosystem. It showed that at 2 h after i.v. injection, the accumulation of Cy7‐labeled c(RGDfK)‐ppGO in PC‐3 tumors was around sevenfold higher than that of the nontargeted control nanoparticles (**Figure** **5**A,B), and this increased distribution pattern maintained for the study duration (24 h after injection) (Figure 5, and Figure S6, Supporting Information). Furthermore, free c(RGDfK) peptides could almost completely block the improved distribution, indicating the dominant contribution of the ligands to the targeting property of the nanosystem. Oku and co‐workers previously have revealed that tumor vessel‐targeted liposomal VP (APRPG‐PEG‐Lip BPD‐MA) at 3 h after i.v. injection already accurately targeted and accumulated in the tumor vascular ECs, conferred dramatically enhanced distribution in tumor sites, and earned superior antitumor efficacy through the VTP compared to the nontargeted ones.^38^ Given the remarkably increased intratumoral distribution of our c(RGDfK)‐modified nanosystem at 2 h after injection, and that the reported similar duration (3 h) after injection has been proved significantly effective in liposomal VP‐mediated VTP, we then selected 2 h after nanoparticle injection for following in vivo VTP and antitumor tests. The targeted distributions of the three loaded cargoes (VP, AQ4N, and siHIF‐1α) were also identified (Figures S7–S9, Supporting Information).

### 2.5 Targeted Trimodal Nanosystem with 690 nm Irradiation Decreased Functional Tumor Vessels and Tumor Perfusion

**Figure 6:**
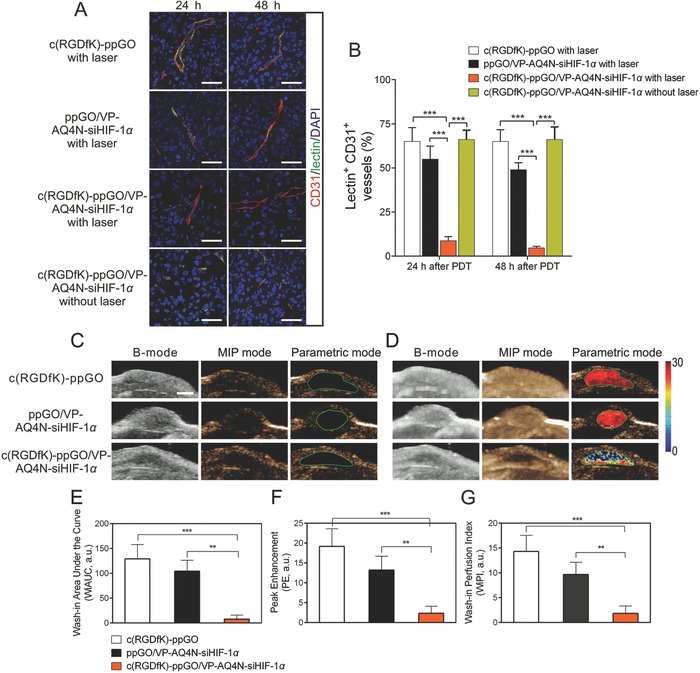
Targeted trimodal nanosystem with 690 nm irradiation resulted in decreased functional vessels and tumor perfusion. A) Tumor vessels were stained by i.v. injection of FITC‐labeled lectin (green) to mark perfused vessels, and the tumor sections were stained with CD31 antibodies (red) to mark all vascular structures. Bar, 50 µm. B) Statistical assay of the percent of lectin^+^CD31^+^ functional vessels compared to whole tumor vessels. Data are presented as mean ± s.d. (*n* = 3) ***p < 0.001. Tumor vascular perfusion was examined using the Vevo 2100 micro‐ultrasound imaging system at 24 h after irradiation. The perfusion images at the baseline C) before the injection of contrast agent and D) at the moment of the strongest contrast signal were shown in three different visualization display. B‐mode (brightness mode) was acquired as original data.Maximum intensity persistence (MAP) mode showed the microvascular network distribution. Pseudo‐color parametric mode displayed the intensity of perfusion kinetics. Tumors are outlined in green circles. Bar, 2.5 mm. Three representative parameters, E) Washing‐in Area Under the Curve (WiAUC), F) Peak Enhancement (PE), and G) Wash‐in Perfusion Index (WiPI) G) were statistically quantified using the Vevo LAB 1.7 software. Data are presented as mean ± s.d. (*n* = 3) **p < 0.01, ***p < 0.001.

It has been well established that VTP can cause tumor vessel occlusion and blood stasis.^2^, ^39^ We then carefully characterize the proportion of functional vessels and the profiles of vascular perfusion in tumors after VTP. The functional vessels were detected using the FITC‐labeled lectin (green) (**Figure** **6**A). This probe can well bind to the complex‐type *N*‐glycans glycoproteins, particularly the poly‐*N*‐acetyllactosamine residues of complex carbohydrates of the endothelial plasmalemma.^40^ The tumor sections were also stained with CD31 antibodies (red) to mark all the vascular structures (Figure 6A). At 24 h after light irradiation, the proportions of lectin^+^CD31^+^ functional vessels was ≈9% in the tumors treated with the targeted trimodal nanosystem, in contrast to 55% with the nontargeted nanosystem (ppGO/VP‐AQ4N‐siHIF‐1α) and 65% with the empty vehicle (c(RGDfK)‐ppGO) (Figure 6A,B). Such significant difference still maintained at 48 h. In sharp contrast, the functional tumor vessels were well maintained if no irradiation was given (Figure 6A,B). These observations definitely demonstrated that VTP treatment shut down the tumor vessel network.

Tumor perfusion was explored using the Vevo 2100 micro‐ultrasound imaging system (Figure 6C,D). 24 h after light irradiation, the ultrasound signal was significantly decreased in the tumors treated with the targeted trimodal nanosystem, indicating the low perfusion in the tumor sites. Quantification of the perfusion parameters (Wash‐in Area Under the Curve, Peak Enhancement, Wash‐in Perfusion index) showed that the targeted trimodal nanosystem with light irradiation was able to effectively block the tumor perfusion compared to the nontargeted control and empty vehicle (Figure 6E–G). These results were also well consistent with the observations on the functional vessels (Figure 6A,B).

### 2.6 Targeted Trimodal Nanosystem with 690 nm Irradiation Aggravated Tumor Hypoxia, Suppressed HIF‐1α, Upregulated CYP450, and Increased AQ4N Activation into AQ4 in Tumors

**Figure 7:**
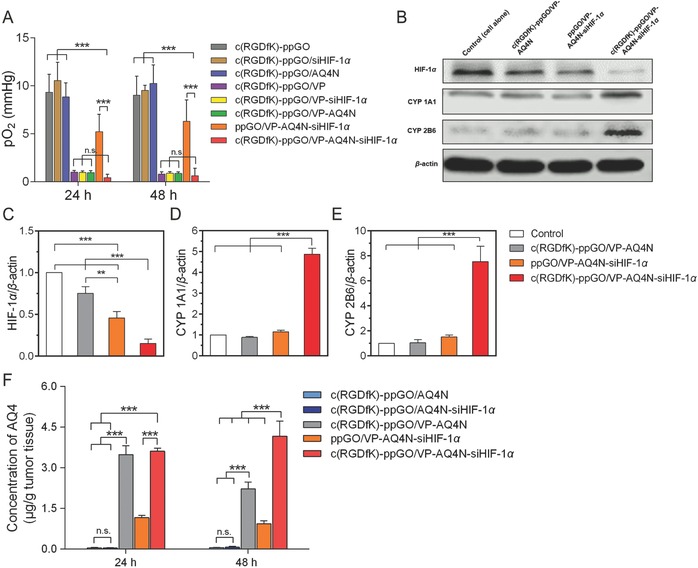
Targeted trimodal nanosystem with 690 nm irradiation decreased tumor pO2, inhibited HIF‐1α expression, upregulated CYP450 expression, and induced more AQ4N activation into cytotoxic AQ4 in tumors. A) Tumor partial oxygen pressure (pO_2_) at 24 and 48 h after irradiation (690 nm, 50 mW cm^−2^, 20 min) was examined using an Oxylite fiber‐optic oxygen sensor. B) 48 h after irradiation, three nude mice from each group were sacrificed. The PC‐3 tumors were excised for Western blot assay of the HIF‐1α, CYP 1A1, and 2B6 proteins. The PC‐3 cells alone under hypoxia were used as control. C–E) Statistical analysis of the relative expression level of the three proteins in tumors. Data are presented as mean ± s.d. (*n* = 3) **p < 0.01, ***p < 0.001. F) The AQ4 concentrations in tumors were determined using LC‐MS/MS method at 24 and 48 h after irradiation. Data are presented as mean ± s.d. (*n* = 3) ***p < 0.001. n.s., not significant.

We then investigated if the occlusion of the functional vessels and declined tumor perfusion after irradiation can lead to aggravated hypoxia. Tumor pO_2_ at 24 and 48 h after irradiation was examined using an Oxylite fiber‐optic oxygen sensor (**Figure** **7**A). The intact PC‐3 tumors at the used size were hypoxic (pO_2_ 8.7 mmHg), which was in sharp contrast with the significantly higher oxygen level (normoxia) in the muscle of mice hind legs (31.0 mmHg) (Figure S10, Supporting Information). It showed tumor pO_2_ remained high around 10 mmHg in the absence of VP, and siHIF‐1α or AQ4N treatment did not influence the pO_2_. However, the pO_2_ declined to below 1 mmHg when VP was involved in the targeted c(RGDfK)‐modified nanoparticles. The pO_2_ was 0.4 mmHg in the group of targeted trimodal nanosystem, in contrast to 5.2 mmHg for the nontargeted control (ppGO/VP‐AQ4N‐siHIF‐1α) and 9.3 mmHg for empty vehicle (c(RGDfK)‐ppGO) at 24 h postirradiation. The pO_2_ in the tumors treated with targeted trimodal nanosystem maintained very low at 48 h (0.6 mmHg), conferring a long‐lasting hypoxic condition for AQ4N activation.

Next, we examined the expression of HIF‐1α, and CYP1A1 and 2B6 in tumors using Western blot (Figure 7B–E). It showed that the HIF‐1α expression in the group of targeted trimodal nanosystem was remarkably suppressed to ≈30%, 20%, and 15% level of those of the nontargeted control, targeted nanosystem without siHIF‐1α (c(RGDfK)‐ppGO/VP‐AQ4N), and the intact hypoxic PC‐3 cells, respectively (Figure 7B,C). Most importantly, the strong inhibition of HIF‐1α led to 4–7.5‐fold higher contents of CYP1A1 or 2B6 proteins compared to all the controls (Figure 7B,D,E). This change would be very helpful for AQ4N activation.

To investigate if such favorable changes in pO_2_ and CYP450 activating enzymes in tumors can strengthen AQ4N activation in vivo, we quantified the intratumoral AQ4 concentrations using the established HPLC‐MS/MS method (Figure 7F, and Figure S11, Supporting Information). Treatment without VP involved, such as c(RGDfK)‐ppGO/AQ4N and c(RGDfK)‐ppGO/AQ4N‐siHIF‐1α, only conferred extremely low AQ4 contents (<0.1 µg g^−1^) in tumors, which was in sharp contrast to any other treatments containing VP, demonstrating the importance of VP‐mediated VTP for hypoxia induction and AQ4N activation (Figure 7A,F). The positive effect of siHIF‐1α on AQ4N activation in tumors was also observed. At 48 h after irradiation, almost twofold AQ4 concentration was found in the tumors treated with the targeted trimodal nanosystem compared to that of control without siHIF‐1α (c(RGDfK)‐ppGO/VP‐AQ4N) (Figure 7F). Such effect of siHIF‐1α can be ascribed to its role of upregulating CYP1A1 and 2B6 prodrug‐activating enzymes (Figure 7B–E). It is also noticed that at 24 h after irradiation, no more AQ4 in tumors was obtained in the group of targeted trimodal nanosystem (Figure 7F), which may be ascribed to the moderate changes in the HIF‐1α inhibition and CYP450 upregulation in this duration (Figure S12, Supporting Information).

The targeted trimodal nanosystem resulted in 3.1‐ and 4.5‐folds higher AQ4 concentrations in tumors compared to nontargeted ppGO/VP‐AQ4N‐siHIF‐1α, at 24 and 48 h after irradiation (Figure 7F). These results were well related with the observation of pO_2_ and CYP1A1 and 2B6 contents in the tumor sites at the same time (Figure 7A–E). Besides, the superior distribution mediated by the c(RGDfK) peptides of the targeted nanosystem compared to that of the nontargeted control may also contribute to the higher AQ4 contents in tumors (Figure 5).

### 2.7 Anticancer Effect of Targeted Trimodal Nanosystem in PC‐3 Tumor‐Bearing Mice

**Figure 8:**
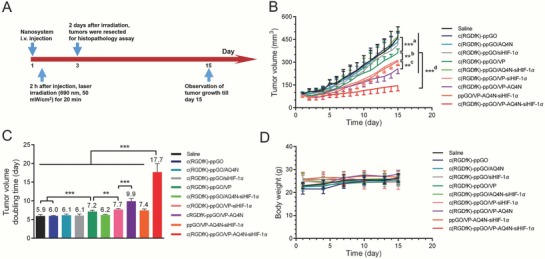
Trimodal nanosystem delayed PC‐3 tumor growth without causing loss of body weight. A) Schematic regimen of the treatment with various nanosystems. At day 1, 2 h after nanoparticle injection, the tumors were irradiated with laser only once (690 nm, 50 mW cm^−2^, 20 min). Then, tumor sizes and mice body weight were monitored for 2 weeks till day 15. B) Tumor growth profiles till day 15. a) c(RGDfK)‐ppGO/VP versus saline and c(RGDfK)‐ppGO. b) c(RGDfK)‐ppGO/VP‐siHIF‐1α versus c(RGDfK)‐ppGO/VP. c) c(RGDfK)‐ppGO/VP‐AQ4N versus c(RGDfK)‐ppGO/VP‐siHIF‐1α. d) c(RGDfK)‐ppGO/VP‐AQ4N‐siHIF‐1α versus all other groups. C) Tumor volume doubling time (DT) was noted on the top of each column. D) Mice body weight throughout the study. The data are presented as mean ± s.d. (*n* = 5) **p < 0.05, ***p < 0.001.

We then evaluated the anticancer efficacy of the targeted trimodal nanosystem in PC‐3 tumor‐bearing mice (**Figure** **8**A). In this study, we carefully set complete controls, including the c(RDGfK)‐modified nanosystem loading VP, AQ4N or siHIF‐1α alone or their pair combinations. Saline, empty vehicle (c(RGDfK)‐ppGO), and nontargeted control (ppGO/VP‐AQ4N‐siHIF‐1α) were also included. Beside tumor growth profiles (Figure 8B), we further quantified the doubling time (DT) of tumor volume (Figure 8C).

It showed nanosystem with VP alone could obviously extend the DT (7.2 d) compared to saline (DT 5.9 d) and c(RGDfK)‐ppGO (empty nanocarrier, DT 6.0 d), demonstrating the potency of VTP in cancer treatment. However, AQ4N alone (DT 6.1 d) was moderately effective at the tested dose, which may be due to its extremely low activation under the relatively high pO_2_ in tumors (Figure 7A,F). siHIF‐1α alone (DT 6.1 d) only displayed slight effect compared to saline. This may be ascribed to the relatively high pO_2_ (Figure 7A), thus low hypoxic stress pressure, which was not a suited circumstance for siHIF‐1α to exhibit its effect.

In the dual‐drug combination treatments, AQ4N plus siHIF‐1α still conferred moderate effect (DT 6.2 d); this can be explained by the respective weak effects of the two molecules as discussed above. However, siHIF‐1α plus VP significantly delayed the tumor growth and increased the DT (7.7 d) compared to the VP or siHIF‐1α alone. This result suggested that the role of siHIF‐1α in inhibiting tumor growth via knocking down HIF‐1α would be more obvious under aggravated hypoxic stress condition, such as that induced by VP‐mediated VTP (Figure 7A). VP plus AQ4N led to dramatically enhanced anticancer effect with DT of 9.9 d, exhibiting the great contribution of AQ4N in delaying tumor growth when exposed under the severe hypoxia induced by VP‐mediated VTP (Figure 7A).

As expected, the targeted VP, AQ4N, and siHIF‐1α combination (trimodal nanosystem) earned the best effect of delaying tumor growth and extending the DT compared to all the single‐drug or dual‐drug treatments. The resulted DT (17.7 d) was 2.9‐, 2.3‐, and 1.8‐folds longer than that of AQ4N plus siHIF‐1α, VP plus siHIF‐1α, and VP plus AQ4N, respectively. Such advantage can be ascribed to the mechanism‐based interlocking effects of the three molecules as previously discussed in the in vitro and in vivo tests, and as illustrated in Figure 1. Furthermore, such extended DT was 2.4‐folds longer than that of nontargeted three‐drug combination (DT 7.4 d). This can be ascribed to the c(RGDfK) ligands, which conferred the targeted nanoparticle distribution in tumor sites (Figure 5) and improved nanoparticle intracellular uptake (Figure 2F,G) to enhance the effects of the three molecules. Moreover, this dramatically enhanced antitumor efficacy of the targeted trimodal nanosystem was not accompanied with overt toxicity such as loss of body weight (Figure 8D).

Histopathological examination showed that the targeted trimodal nanosystem led to much more karyopyknosis and karyorrhexis, the morphological features of apoptosis,^41^ in the tumor cells (Figure S13A,B, Supporting Information). Moreover, such treatment resulted in much more TUNEL‐positive cells (Figure S13C,D, Supporting Information) and less PCNA‐positive cells (Figure S13E,F, Supporting Information) in tumors compared to those of all the other controls.

## 3 Discussion

Cancer combination therapy can be clinically beneficial due to the comprehensive effects of increasing therapeutic efficacy and lowering the required dose, hence side effects or toxicity.^42^ Hypoxia is one of the hallmarks of solid tumor, and is considered as an important therapeutic target in cancer treatment. Developing strategies to relieve or exploit hypoxia is an emerging attractive field in cancer nanotechnology. To the best of our knowledge, this study presented the first example using VTP, a clinically relevant strategy, to actively induce aggravated hypoxia for enhanced HAP activation and anticancer therapy. However, hypoxia also leads to the downregulation of CYP450 (the dominant HAP‐activating reductases) through the induced HIF‐1α, which eventually compromises the HAP activation efficacy. This important hidden negative effect of hypoxia on CYP450 and HAP activation has not ever been considered in previous reports, and this issue is highlighted and well resolved in our research. All these were successfully accomplished with the engineered targeted trimodal graphene‐oxide‐based nanosystem (c(RGDfK)‐ppGO/VP‐AQ4N‐siHIF‐1α).

Restrained HAP activation in vivo is often encountered due to the heterogeneous TME, which cannot offer required low pO_2_.[[qv: 13a]] We here developed a new method to promote HAP activation by actively inducing hypoxia via VTP. This was achieved by using c(RGDfK)‐modified VP‐loaded nanoparticle that can target integrin α_v_β_3_ on tumor vascular ECs. As a facile in vitro model, tumor spheres have been used to evaluate the effects of drug and nanoparticles on cancer therapy.^43^ However, there is so far no such model that involves both tumor cells and vascular system with perfused blood. Therefore, we focused the in vivo tests to carefully investigate the influence of VP on AQ4N activation. It showed that VP‐based VTP in vivo led to serious blood occlusion, which was proved in the observation of dramatically decreased functional tumor vessels and perfusion (Figure 6). In vitro VTP with HUVECs as tumor vascular EC model also confirmed the targeted damage to tumor vascular ECs (Figure 3A–C). Such antivascular effects and induced aggravated hypoxia (Figure 7A) compelled much more AQ4N activation into AQ4, which was shown when comparing the intratumoral AQ4 contents between the nanosystem containing AQ4N alone and the one carrying both VP and AQ4N (Figure 7F).

However, hypoxic stress also leads to high expression of HIF‐1α, which acts as “hidden brake” to substantially hinder AQ4N activation through downregulating CYP450 activating reductases. Here, we adopted siHIF‐1α to effectively suppress HIF‐1α, therefore increase CYP450 in hypoxic tumor cells, and further enhance AQ4N activation. The influence of siHIF‐1α on AQ4N was evaluated both in vitro and in vivo. It showed that siHIF‐1α significantly enhanced the cytotoxicity of AQ4N to hypoxic PC‐3 cells, although siHIF‐1α alone was nontoxic to the cells (Figure 4G). This effect of siHIF‐1α can be related with its role of upregulating the AQ4N‐activating reductases (CYP1A1 and 2B6) (Figure 4B–F). As AQ4N activation depends on both hypoxia and reductase (CYP450) (Figure 4H),[[qv: 37b]] the influence of siHIF‐1α on in vivo AQ4N activation presented VP‐dependent pattern. Without VP‐mediated VTP, siHIF‐1α could not increase the AQ4N activation in tumors (Figure 7F), as the relatively high pO_2_ (less hypoxia) in the tumors already seriously hindered AQ4N activation (Figure 7F). However, with the involvement of VP‐mediated VTP, the effect of siHIF‐1α on AQ4N activation in tumors could be obviously observed. At 48 h, nearly twofold AQ4 concentration was obtained in the tumors treated with the targeted trimodal nanosystem compared to that of control without siHIF‐1α (c(RGDfK)‐ppGO/VP‐AQ4N) (Figure 7F). As siHIF‐1α did not confer more hypoxia in this scenario (48 h after irradiation) (Figure 7A), such effect of siHIF‐1α may be mainly ascribed to its role of upregulating CYP450 reductases in that duration, through HIF‐1α inhibition (Figures 4B–F and 7B–E), for strengthened AQ4N activation in tumors.

Taken together, these data demonstrated that both VP and siHIF‐1α can effectively promote the efficacy of AQ4N, warranting a successful trimodal anticancer therapy. It should be noted that although the trimodal nanosystem was designed mainly to target the tumor vessels, the treatment may not be restricted to only perivascular tissues. AQ4N with its high penetrating property can spread deeply into the tumor tissue for enhanced drug exposure and killing of the hypoxic tumor cells in the inner region.^44^ Moreover, it showed that HIF‐1α expression in PC‐3 tumors treated with the targeted trimodal nanosystem (with siHIF‐1α) was remarkably suppressed to ≈20% level of that treated with the targeted nanosystem without siHIF‐1α (c(RGDfK)‐ppGO/VP‐AQ4N) (Figure 7B,C). This observation indicated that siHIF‐1α delivered by the nanosystem can exert its function (HIF‐1α suppression) in a wider tumor region. Furthermore, VTP‐induced exacerbated hypoxia in the region distant from the vessels can directly kill the tumor cells and lead to tumor regression, which is the major rationale for VTP to treat solid tumors in clinic.^2^, ^45^ The histopathological examination also demonstrated that the targeted trimodal nanosystem resulted in widespread apoptosis in the tumors (Figure S13, Supporting Information).

## 4 Conclusion

In conclusion, we have developed an interlocking trimodal graphene‐oxide‐based nanosystem to improve VTP, by targeting tumor hypoxia with HAP (AQ4N) and siHIF‐1α. Meanwhile, our study offers a new way to enhance HAP efficacy, which is achieved by nanoparticle‐mediated VTP to induce aggravated hypoxia and siHIF‐1α intervention to upregulate the expression of CYP450 (the key HAP‐activating reductases). Our therapeutic strategies present promising nanomaterial‐based approaches towards the clinical applications of both VTP, which was recently approved for solid tumor,[[qv: 4b]] and HAPs, which are experiencing bottleneck in clinical trials due to the compromised prodrug activation under low levels of tumor hypoxia in some clinical settings.[[qv: 13b]]

## 5 Experimental Section

*Materials, Cell Culture, and Animals*: AQ4N was purchased from Tocris Bioscience (Bristol, UK). AQ4, branched PEI (MW 25 kDa), EDC, NHS, suberic acid bis (3‐sulfo‐N‐hydroxysuccinimide ester) sodium salt (BS^3^), FITC conjugated lectin from *Bandeiraea simplicifolia*, NADPH, ethidium bromide, and 4′,6‐diamidino‐2‐phenylindole dihydrochloride (DAPI) were from Sigma‐Aldrich (St. Louis, MO). VP was supplied by Selleck (Shanghai, China). Graphene was purchased from JCNANO (Nanjing, China). Eight‐armed amine‐terminated polyethylene glycol (PEG, MW 10 kDa) was supplied from JenKem (Beijing, China). Cy7‐NHS was from Bridgen (Beijing, China). c(RGDfK) peptide was synthesized by GL Biochem (Shanghai, China). FAM‐labeled siRNA targeting HIF‐1α (siHIF‐1α, antisense strand, 5′‐UGUAGUAGCUGCAUGAUCGdTdT‐3′) and negative control siRNA with a scrambled sequence (siNC, 5′‐GACUACUGGUCGUUGAACU dTdT‐3′) were synthesized by GenePharma (Shanghai, China). Rabbit anti‐HIF‐1α antibody was supplied by Cell Signaling Technology (Danvers, MA). Rabbit anticytochrome P450 1A1 and 2B6 antibodies were purchased from Abcam (Hong Kong). Rabbit antimouse CD31 antibody and Rat Pooled Liver Microsomes Male were purchased from BD Biosciences (San Jose, CA). Double distilled water was purified using a Millipore simplicity system (Millipore, Bedford, MA). All other chemicals were of analytical grade and used without further purification. A fiber coupled laser diode with 640 mW output at 690 nm (Inter‐Diff Optoelectronics, Shanghai, China) was used. The exact optical powers of lasers used in this study were corrected and recorded by a 690 nm laser energy meter (Inter‐Diff Optoelectronics, Shanghai, China).

Primary HUVECs and M200 medium with LSGS were obtained from Life Technologies (Carlsbad, CA). Cells at 3–5 passages were used in the experiments. Human PC‐3 prostate cancer cell line was obtained from the American Type Culture Collection (Manassas, VA) and cultured in DMEM/F‐12 medium (Gibco, Life Technology) supplemented with 10% FBS and antibiotics (100 mg mL^−1^ of streptomycin and 100 U mL^−1^ of penicillin) at 37 °C in a humidified incubator with 5% CO_2_.

Male BALB/c nude mice (≈20 g) were provided by Shanghai Laboratory Animal Center (Chinese Academy of Sciences, Shanghai, China). The animal experiment designed in this study was approved by the ethical committee of Shanghai Jiao Tong University School of Medicine (SJTU‐SM).

*Preparation and Characterization of Targeted Trimodal Nanosystem (c(RGDfK)‐ppGO/VP‐AQ4N‐siHIF‐1α)*: GO was synthesized according to previously reported modified Hummers method using graphene as the original material.^46^ For the preparation of nanoscale PEGylated GO (PEG‐GO, pGO), the GO solution (1 mg mL^−1^) was mixed with eight‐armed NH_2_‐terminated PEG (3 mg mL^−1^) and sonicated for 5 min. Then, EDC (1 mg mL^−1^) was added to the mixture for 30 min sonication. After that, the mixture was added with NHS (1.2 mg mL^−1^) for another 5 min sonication, and was stirred gently at room temperature (RT) for 12 h. Then the mixture was washed 3–5 times with deionized water using 100 KDa Milli‐Q membrane filter (Millipore, Bedford, MA) (4000 r min^−1^, 10 min), obtaining the pGO re‐suspended in water.

pGO in water was sequentially mixed with verteporfin (VP, 0.75 mg mL^−1^) and AQ4N (1 mg mL^−1^) and stirred at RT for 12 h, respectively. The obtained pGO/VP‐AQ4N was stirred at RT for another 30 min following the addition of c(RGDfK) peptide (0.4 mg mL^−1^) and BS^3^ (3.5 mg mL^−1^). After 3–5 times washing, the c(RGDfK)‐pGO/VP‐AQ4N mixture was sonicated with PEI (5 mg mL^−1^) for 5 min. Then, EDC was added at final concentration of 2.5 mg mL^−1^, and the mixture was stirred at RT for 6 h. Then, the resulted c(RGDfK)‐ppGO/VP‐AQ4N mixture was washed five times and re‐suspended.

To study HIF‐1α siRNA (siHIF‐1α) loading on the c(RGDfK)‐ppGO/VP‐AQ4N complexes, a gel electrophoresis assay was performed after incubation of c(RGDfK)‐ppGO/VP‐AQ4N with siHIF‐1α at different N/P ratios (2.5, 5, 10, 20, 40, 80, and 120). Different amounts of the c(RGDfK)‐ppGO/VP‐AQ4N solution were mixed with 800 ng of the siHIF‐1α solution in equal volume, followed by incubation for 30 min at RT. The complexes were electrophoresed in 1% (w/v) agarose containing ethidium bromide (0.5 µg mL^−1^) with TAE buffer (Tris‐acetate‐EDTA) at 120 v for 30 min. The gel was imaged by an Odyssey Fc Image System (LI‐COR, Lincoln, NE) to identify the optimal N/P ratio.

For the preparation of empty ppGO (PEI‐PEG‐GO), c(RGDfK)‐ppGO, c(RGDfK)‐ppGO loading only one or two molecules, and nontargeted trimodal nanosystem (ppGO/VP‐AQ4N‐siHIF‐1α), the preparation procedure was same to that for the targeted trimodal nanosystem except the addition of VP, AQ4N, PEI, or c(RGDfK) if it was required. The concentrations of GO, pGO, and ppGO were quantified by their absorbance at 230 nm as previously reported.^46^ The contents of PEI and PEG in the nanocarrier (ppGO) were also quantified. PEI was assayed using the cuprammonium complex method.^29^ The PEG content was determined using the PEGylated protein ELISA kit (Enzo Life Sciences, Farmingdale, NY).^30^ The c(RGDfK) assay was performed using the CBQCA Protein Quantitation Kit (Thermo Fisher Scientific, Shanghai, China),^31^ then the c(RGDfK) content in the targeted nanocarrier (c(RGDfK)‐ppGO) can be estimated.

The morphology of the GO‐based nanosystem was observed using AFM. The size and zeta potential of the various nanoparticles was measured with a Zetasizer Nano ZS instrument (Malvern, Worcestershire, UK). Drug loading (DL%) was expressed as the percentage of the drug amount found in the nanoparticles. The content of VP and AQ4N were determined from the absorbance at 410 and 610 nm, respectively in the UV–vis–NIR spectra, after subtracting the absorption contribution of corresponding background according to the literature.^47^ The colloidal stability of nanoparticles was evaluated in pure PBS (pH 7.4, 0.01 m) and PBS with 10% FBS at 37 °C according to the literature,^48^ and the hydrodynamic size of the nanoparticles was examined at 24 and 48 h, respectively (*n* = 3).

*Targeted Uptake of c(RGDfK)‐ppGO/VP in HUVECs and PC‐3 Cells*: For cell uptake examination, HUVECs (a cell model mimicking tumor vascular ECs) and PC‐3 cells were cultured at a density of 5 × 10^3^ cells per well in 96‐well plates, respectively. When the cells reached about 80% confluence, the culture medium was replaced by c(RGDfK)‐ppGO/VP or ppGO/VP in medium at nanoparticle concentration of 10 µg mL^−1^ or 50 µg mL^−1^ in 200 µL for 2 h, respectively. After removing the nanoparticles and washing the wells three times with PBS, the cells were fixed by 4% formaldehyde solution for 15 min, and the cell nuclei were stained with 0.1 µg mL^−1^ DAPI for 8 min. Then, cellular uptake was observed and assayed by quantifying the intracellular fluorescence intensity of VP (Ex: 650 nm, Em: 690 nm) on the Thermo Scientific ArrayScan XTI High Content Analysis (HCA) Reader.^49^ The quantitative results were acquired based on 15 random microscope fields in each well, and the tests were replicated for four times.

*Cell Viability after Photodynamic Treatment with c(RGDfK)‐ppGO/VP In Vitro*: Briefly, HUVECs and PC‐3 cells were cultured at a density of 5 × 10^3^ cells per well in 96‐well plates, respectively. After 24 h incubation at 37 °C, the culture medium was replaced by 200 µL of c(RGDfK)‐ppGO/VP or ppGO/VP in medium at VP dose of 0.001 × 10^−6^, 0.01 × 10^−6^, 0.1 × 10^−6^, and 1 × 10^−6^ m for 6 h, respectively. Then, cells were irradiated by the 690 nm laser for 10 min at the power density of 30 mW cm^−2^, and cultured in fresh medium for another 24 h. Then the cell viabilities were quantitatively determined using Cell Counting Kit‐8 (CCK‐8) assay.^49^ Also the cell viabilities were qualitatively evaluated using LIVE/DEAD cell viability/cytotoxicity kit (Life technology, Carlsbad, CA).^50^ In this assay, calcein‐AM is enzymatically converted into green fluorescent calcein in live cells, while ethidium homodimer stains the nuclei of dead cells with red fluorescence. After irradiation treatments and culture for another 24 h, the medium was replaced with 1 mL PBS containing 0.5 µg mL^−1^ calcien‐AM (Ex: 488 nm and Em: 515 nm) and 5 µg mL^−1^ ethidium homodimer (Ex: 535 nm and Em: 615 nm), to stain live and dead cells. Then, the cells were photographed under the microscope. The tests were replicated for three times. ADMA was also used to detect the production of singlet oxygen and verify the PDT performance.^33^

*Lysosomal Escape of c(RGDfK)‐ppGO/FAM‐siHIF‐1α*: Escape from the lysosomes is important for effective delivery of siRNA into the cytosol. PC‐3 cells were incubated with c(RGDfK)‐ppGO/FAM‐siHIF‐1α (green, Ex: 488 nm, Em: 520 nm) for 0.5–4 h, respectively. siHIF‐1α at 150 × 10^−9^ m was used in this test. The lysosomes were labeled with LysoTracker Red DND‐99 (Life technology, Carlsbad, CA) (red, Ex: 577 nm, Em: 590 nm). Time‐dependent intracellular trafficking of c(RGDfK)‐ppGO/FAM‐siHIF‐1α was detected under CLSM (LSM‐510, Carl Zeiss AG, Oberkochen, Germany).

*Expression of HIF‐1α, CYP1A1, and CYP2B6 in Tumor Cells and Xenograft*: Protein expressions were examined using western blot. For in vitro assay, PC‐3 cells were seeded in 6‐well plates (1 × 10^5^ cells per well) and incubated for 24 h (normoxia, 5% CO_2_). Then the cells were incubated with c(RGDfK)‐ppGO/siHIF‐1α, and other controls including ppGO/siHIF‐1α, empty c(RGDfK)‐ppGO, and c(RGDfK)‐ppGO/siNC for 12 h under hypoxia (1% O_2_, 5% CO_2_ balanced with N_2_), respectively (*n* = 3). siHIF‐1α at 150 × 10^−9^ m was used in all the groups with the molecule involved. For in vivo assay, 48 h after the PDT treatment (690 nm, 50 mW cm^−2^, 20 min), three nude mice from each group (c(RGDfK)‐ppGO/VP‐AQ4N‐siHIF‐1α, c(RGDfK)‐ppGO/VP‐AQ4N, and ppGO/VP‐AQ4N‐siHIF‐1α) were sacrificed, and tumors were excised. Cells alone under hypoxia were used as control.

Protein extraction from cells or homogenized tumors was performed using RIPA Lysis Buffer supplemented with Complete Protease Inhibitor Cocktail Tablets (Roche, Rotkreuz, Switzerland).^51^ The protein concentrations were determined with BCA Protein Assay Kit (Thermo Scientific, Rockford, IL). Equivalent amount (30 µg) of protein from different samples were applied to 10% SDS‐PAGE, and then electrically transferred (220 mA, 120 min) onto Immobilon‐P membranes (Millipore, Bedford, MA). Then, the membranes were blocked in tris‐buffered saline (TBS) with 0.05% Tween‐20 (TBST) containing 5% bovine serum albumin (BSA) for 1 h at RT, and then incubated in TBST containing 1% BSA and primary antibody against HIF‐1α (1:1000), CYP1A1 (1:1000) and CYP2B6 (1:1000) overnight at 4 °C, respectively. Then, the membranes were washed thrice with TBST and incubated with horseradish peroxidase‐conjugated goat antirabbit antibody (1:5000, Cell Signaling Technology, Danvers, MA) for 1 h at RT. Finally, the membranes were imaged by an Odyssey Fc Image System (LI‐COR, Lincoln, NE).

*AQ4N Toxicity to PC‐3 Cells in the Presence of siHIF‐1α and Exogenous CYP450 from Liver Microsomes*: PC‐3 cells were cultured at a density of 5 × 10^3^ cells per well in 96‐well plates. After 24 h incubation at 37 °C, the cells were incubated with c(RGDfK)‐ppGO nanoparticles carrying both AQ4N (0.4 × 10^−6^ and 2 × 10^−6^ m) and siHIF‐1α (150 × 10^−9^ m) under hypoxia (1% O_2_, 5% CO_2_ balanced with N_2_) for 4 h, and then the cells were rinsed with PBS and incubated in normoxia (95% air, 5% CO_2_) for another 24 h. The nanoparticles carrying AQ4N or siHIF‐1α alone, and the empty vehicle (c(RGDfK)‐ppGO) were also included in the tests. The PC‐3 cells alone under hypoxia was set as control. The cell viabilities were detected using CCK‐8 assay. The tests were replicated for three times.

Also, PC‐3 cells were treated with c(RGDfK)‐ppGO or ppGO nanoparticles carrying AQ4N (0.08 × 10^−6^, 0.4 × 10^−6^, and 2 × 10^−6^ m) and exposed under normoxia or hypoxia with/without CYP450‐contained Rat Pooled Liver Microsomes Male (3 mg mL^−1^) plus NADPH (5 × 10^−3^ m) for 4 h. Then, the cells were rinsed with PBS and incubated in normoxia for another 24 h. The PC‐3 cells alone under normoxia was set as control. The cell viabilities were detected using CCK‐8 assay. The tests were replicated for three times.

*In Vivo Tumor Targeting of the Nanosystem in PC‐3 Xenograft*: Cy7‐NHS was conjugated to the amines of PEI of c(RGDfK)‐ppGO. The real‐time tumor accumulation of Cy7‐labeled c(RGDfK)‐ppGO in male BALB/c nude mice bearing subcutaneous PC‐3 xenografts (150 mm^3^) on the right hind limb were monitored under the Xenogen IVIS 200 (Caliper Life Sciences, MA) imaging system.^52^ Three mice from each group were injected through the caudal vein with 0.4 mg kg^−1^ of Cy7‐labeled c(RGDfK)‐ppGO or ppGO, respectively. After 1, 2, 8, and 24 h, the mice were anaesthetized and imaged with an excitation bandpass filter at 750 nm and an emission at 790 nm. The exposure time for each image was 3 s. The blocking effect of 50‐fold molar excess c(RGDfK) on the tumor tissue targeting was also evaluated.

The targeted distribution of the loaded three active molecules (VP, AQ4N, and siHIF‐1α) in tumors was also determined. The nanoparticles (ppGO/VP‐AQ4N‐siHIF‐1α and c(RGDfK)‐ppGO/VP‐AQ4N‐siHIF‐1α; VP 1 mg kg^−1^, AQ4N 5 mg kg^−1^, and Cy7‐labeled siHIF‐1α 0.5 mg kg^−1^) were i.v. injected to the mice bearing PC‐3 tumor (*n* = 3). After 2 h, the accumulation of Cy7‐labeled siHIF‐1α in tumors were examined using the Xenogen IVIS 200 imaging system as described in the literature.^53^ For VP and AQ4N assay, the mice were sacrificed, and tumors were excised and homogenized. Protein precipitation was produced with three volumes of acetonitrile (0.1% acetic acid) for AQ4N and VP sample preparation. Analyses were performed on a QTRAP 5500 LC‐MS/MS System (AB Sciex, USA) equipped with Shimazu LC‐20 HPLC system (Shimazu, Japan). A Waters XBridge C18 Column (2.1 mm × 50 mm, 3.5 µm) was used for analyte separation. Gradient elution with a mobile phase consisting of water and acetonitrile (0.1% acetic acid in both components, 0.4 mL min^−1^) was used for the separation. For VP assay, a gradient elution with 50/50 (0–0.5 min), 25/75 (0.5–1 min), 5/95 (1–4.5 min) and 50/50 (4.5–7.5 min) (water/acetonitrile, v/v) composition was used. For AQ4N detection, a gradient elution with 98/2 (0–0.5 min), 5/95 (0.5–2 min), 5/95 (2–5 min), and 98/2 (5–7.5 min) composition was adopted. AQ4N (*m/z* 445.2–323.0, DP 90 V, EP 10 V, CE 30 V), VP (*m/z* 719.2–645.2, DP 90 V, EP 10 V, CE 58 V) and IS (paclitaxel, *m/z* 854.4–286.1, DP 190 V, EP 14 V, CE 21 V) were monitored. Data processing of MS was performed on the software package (AB Sciex, USA).

*Mouse Model and Treatment Protocol*: Male BALB/c nude mice were subcutaneously inoculated with PC‐3 cells (5 × 10^6^ cells per mouse). When the tumors grew to ≈100 mm^3^, the mice were randomized into 10 groups (*n* = 8): (1) saline (Control); (2) empty c(RGDfK)‐ppGO; (3) c(RGDfK)‐ppGO/VP; (4) c(RGDfK)‐ppGO/AQ4N; (5) c(RGDfK)‐ppGO/siHIF‐1α; (6) c(RGDfK)‐ppGO/VP‐AQ4N; (7) c(RGDfK)‐ppGO/VP‐siHIF‐1α; (8) c(RGDfK)‐ppGO/AQ4N‐ siHIF‐1α; (9) ppGO/VP‐AQ4N‐siHIF‐1α; (10) c(RGDfK)‐ppGO/VP‐AQ4N‐siHIF‐1α. VP (1 mg kg^−1^), AQ4N (5 mg kg^−1^), and siHIF‐1α (0.5 mg kg^−1^) were given in all cases involved.

At 2 h after nanoparticle injection, the tumors were irradiated with laser (690 nm, 50 mW cm^−2^) for 20 min 48 h after irradiation, three mice from each group were sacrificed, and the tumors were removed and processed for paraffin sections and histopathological examination. Tumor cell apoptosis and proliferation were identified using ApopTag Peroxidase In situ Apoptosis Detection Kit (Merck Millipore, Billerica, MA) and mouse antihuman antibody against PCNA (Santa Cruz, CA), respectively. Quantitative histopathological assay was performed using Image‐Pro Plus 6.0 software (Media Cybernetics, Bethesda, MD). Tumor sizes (*n* = 5) were monitored with a digital caliper every day throughout the study, and tumor volume was calculated as volume (mm^3^) = length × width^2^/2. In addition, tumor volume DT was calculated with the following equation:^54^ DT =*T* × log 2/(log *V*_F_ – log *V*_i_), where *V*_F_ is the final tumor volume, *V*_i_ is the initial tumor volume at drug treatment time point, and *T* is the time difference between the initial and the final day. The body weights of all mice were measured every 3 d for two weeks till day 15.

*Detection of Functional and Whole Vessels in Tumors*: At 24 and 48 h after irradiation, FITC‐labeled *Bandeiraea simplicifolia* lectin (0.1 mg kg^−1^) was injected into PC‐3 tumor bearing mice via tail vein to characterize functional vessels.^40^ At 20 min after lectin injection, three nude mice from each group (c(RGDfK)‐ppGO, ppGO/VP‐AQ4N‐siHIF‐1α and c(RGDfK)‐ppGO/VP‐ AQ4N‐siHIF‐1α) were sacrificed. The treatment with c(RGDfK)‐ppGO/VP‐AQ4N‐siHIF‐1α but no irradiation was also included as control. The tumors were removed and processed for sections and immunofluorescence staining with rabbit antimouse CD31 antibody (1: 200) for whole tumor vascular ECs.^40^ The secondary antibody was Alexa Flour 647 donkey antirabbit IgG (Life Technologies, Shanghai, China). The slides were observed for tumor total vessels and functional vessels under CLSM for CD31 (Ex: 650 nm, LP Em: 668 nm) and FITC‐labeled lectin (Ex: 495, Em: BP 505–550 nm), respectively. The percentage of lectin and CD31 dual‐positive functional vessels were estimated using Image‐Pro Plus 6.0 software (Media Cybernetics, Bethesda, MD).

*Tumor Vascular Perfusion*: The Vevo 2100 micro‐ultrasound imaging system (FujiFilm VisualSonics, Toronto, Canada) was used to evaluate the tumor vascular perfusion.^55^ Briefly, 24 h after irradiation, three mice from each group (c(RGDfK)‐ppGO, ppGO/VP‐AQ4N‐siHIF‐1α and c(RGDfK)‐ppGO/VP‐AQ4N‐siHIF‐1α) were anaesthetized with the mixture of 3.0% isofluorane and medical air mixture and placed on the warmed platform. The MicroMarker contrast agent (FujiFilm VisualSonics, Toronto, Canada) was prepared with a final concentration of 2 × 10^9^ microbubbles in 1 mL saline solution, and a 50 µL bolus was delivered to the mice via tail vein catheter, and then the image acquisition started. Three representative characteristic parameters (Wash‐in Area Under the Curve, Peak Enhancement, Wash‐in Perfusion Index) describing the speed and extent of the vascular perfusion were calculated by the software Vevo LAB 1.7 (FujiFilm VisualSonics, Toronto, Canada).^49^

*Tumor Oxygenation*: At 24 and 48 h after irradiation, a fiber‐optic oxygen sensor (Oxylite, Oxford Optronix, Abingdon, UK), based on the principle of oxygen quenching of fluorescence, was used for tissue oxygenation pressure (pO_2_) monitoring in the tumors as previously described.^56^ Three mice from each group were tested.

*AQ4N Activation into AQ4 after Treatment with Targeted Trimodal Nanosystem plus Irradiation*: AQ4N can be reductively activated into AQ4 under hypoxic conditions by several cellular enzymes, such as CYP450. At 24 and 48 h after irradiation, three nude mice from each group (c(RGDfK)‐ppGO/AQ4N, c(RGDfK)‐ppGO/AQ4N‐siHIF‐1α, c(RGDfK)‐ppGO/VP‐AQ4N, ppGO/VP‐AQ4N‐siHIF‐1α, c(RGDfK)‐ppGO/VP‐AQ4N‐siHIF‐1α; VP 1 mg kg^−1^, AQ4N 5 mg kg^−1^, and siHIF‐1α 0.5 mg kg^−1^ in all groups with drug involved) were sacrificed, and tumors were excised and homogenized. Protein precipitation was produced with three volumes of acetonitrile for AQ4 sample preparation. Analyses were performed on an Agilent 6410 triple quadrupole mass spectrometer (Agilent Technologies, USA) equipped with electrospray ionization and an Agilent 1200 HPLC system (Agilent Technologies, USA). A Merck ZIC‐HILIC column (2.1 mm × 100 mm, 3.5 µm) was used for analyte separation. Isocratic elution with a mobile phase consisting of acetonitrile and water (60: 40, v/v, the aqueous phase contained 0.1% formic acid and 10 × 10^−9^ m ammonium formate, 0.3 mL min^−1^) was used for the separation. Two MRM transitions, AQ4 (*m/z* 413.2–72.2, fragmentor 140 eV, collision energy 20 eV) and IS (glycyrrhetic acid, *m/z* 471.5–177.1, fragmentor 160 eV, collision energy 30 eV) were monitored. Data processing of MS was performed on the MassHunter software package (VersionB.04.00, Agilent Technologies).

*Statistical Analysis*: Statistical analysis was conducted using GraphPad Prism 6.0 software (La Jolla, CA). Differences between groups were examined using Student's *t*‐test or ANOVA with Tukey's multiple comparison tests. Differences were considered significant if *p* value was less than 0.05.

## Conflict of Interest

The authors declare no conflict of interest.

## Supporting information

Supplementary
