# Supplementary material for: A Tumor Vascular‐Targeted Interlocking Trimodal Nanosystem That Induces and Exploits Hypoxia
Source: Adv Sci (Weinh). 2018 May 28;5(8):1800034. doi: 10.1002/advs.201800034 (PMC6097144; doi:10.1002/advs.201800034)
Supplement: Supplementary file 1 — Supplementary [file ADVS-5-1800034-s001.pdf]

Copyright WILEY-VCH Verlag GmbH & Co. KGaA, 69469 Weinheim, Germany, 2018.

# Supporting Information

for *Adv. Sci.,* DOI: 10.1002/advs.201800034

# A Tumor Vascular-Targeted Interlocking Trimodal Nanosystem That Induces and Exploits Hypoxia

*Xin Luan, Ying-Yun Guan, Hai-Jun Liu, Qin Lu, Mei Zhao, Duxin Sun, Jonathan F. Lovell, Peng Sun, Hong-Zhuan Chen,* and Chao Fang**

# Supporting Information

## A Tumor Vascular-Targeted Interlocking Trimodal Nanosystem that Induces and Exploits Hypoxia

*Xin Luan, Ying-Yun Guan, Hai-Jun Liu, Qin Lu, Mei Zhao, Duxin Sun, Jonathan F. Lovell, Peng Sun, Hong-Zhuan Chen,* and Chao Fang**

**Table S1** Size and zeta potential of various GO-based formulations determined using a Malvern Zetasizer Nano ZS instrument. Values are expressed as mean ± s.d. (n=3)

| | Size (nm) | Zeta potential (mV) |
|---|---|---|
| GO | 79.9 ± 5.6 | - 37.5 ± 1.6 |
| pGO (PEG-GO) | 84.7 ± 2.7 | -21.5 ± 0.9 |
| ppGO (PEI-PEG-GO) | 89.6 ± 3.1 | 24.8 ± 0.6 |
| c(RGDfK)-ppGO | 88.7 ± 2.4 | 22.3 ± 1.0 |
| c(RGDfK)-ppGO/VP | 89.9 ± 1.6 | 21.9 ± 0.4 |
| c(RGDfK)-ppGO/AQ4N | 90.1 ± 2.0 | 22.1 ± 0.7 |
| c(RGDfK)-ppGO/siHIF-1$\alpha$ | 88.4 ± 1.3 | 19.1 ± 0.6 |
| c(RGDfK)-ppGO/VP-AQ4N | 91.3 ± 3.6 | 20.9 ± 1.1 |
| c(RGDfK)-ppGO/VP-siHIF-1$\alpha$ | 90.9 ± 2.5 | 18.7 ± 0.9 |
| c(RGDfK)-ppGO/AQ4N-siHIF-1$\alpha$ | 89.7 ± 1.2 | 19.9 ± 0.8 |
| ppGO/VP-AQ4N-siHIF-1$\alpha$ | 90.5 ± 3.2 | 17.4 ± 0.2 |
| c(RGDfK)-ppGO/VP-AQ4N-siHIF-1$\alpha$ | 91.3 ± 2.4 | 16.2 ± 0.3 |

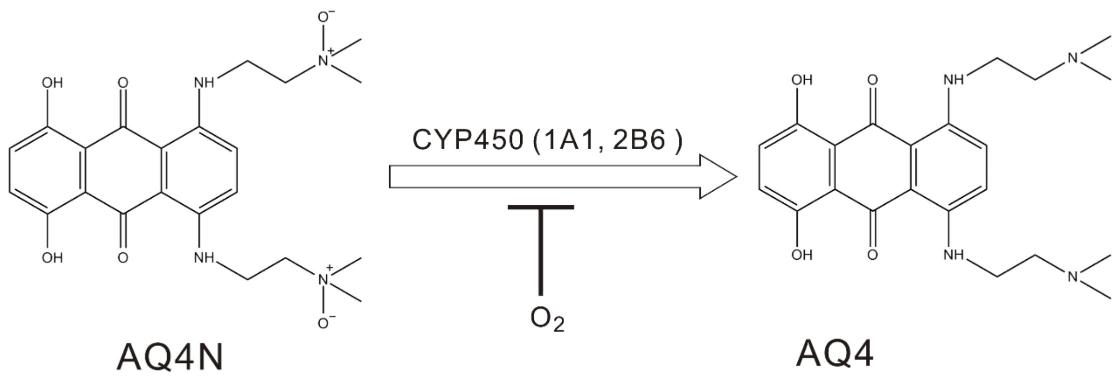


**Figure S1. The mechanism of AQ4N activation into AQ4.** The activation of AQ4N depends on both hypoxia and CYP450 (1A1 and 2B6) activating reductases in tumors, which metabolize AQ4N into AQ4 (a potent inhibitor of topoisomerase II). However, oxygen blocks this process through outcompeting AQ4N for haem-centred active site of CYP450.

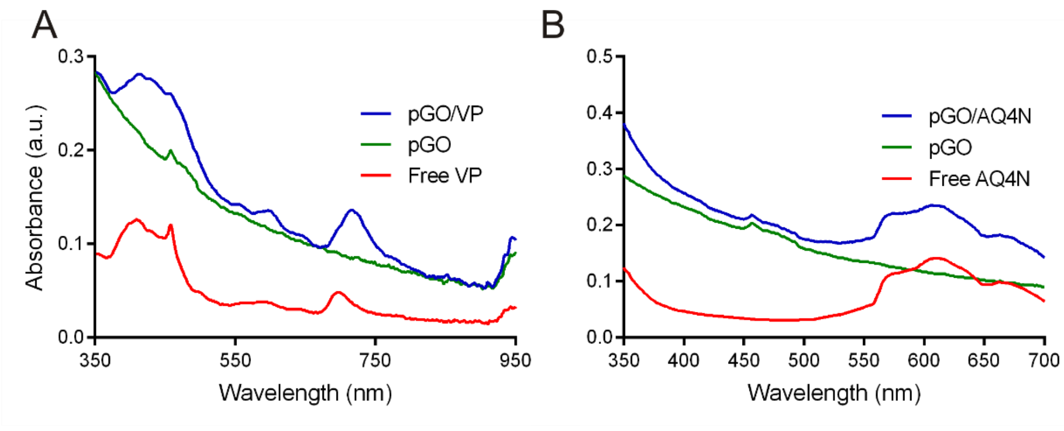


**Figure S2. Identification of VP and AQ4N loaded on the GO-based nanoparticles.** The loadings of VP and AQ4N on the nanoparticles were identified with UV-vis-NIR absorbance detection. The absorbance peaks were at 410 nm for VP and 610 nm for AQ4N. (**A**) UV-vis-NIR absorbance spectrum of VP-loaded pGO (pGO/VP). (**B**) UV-vis absorbance spectrum of AQ4N-loaded pGO (pGO/AQ4N).

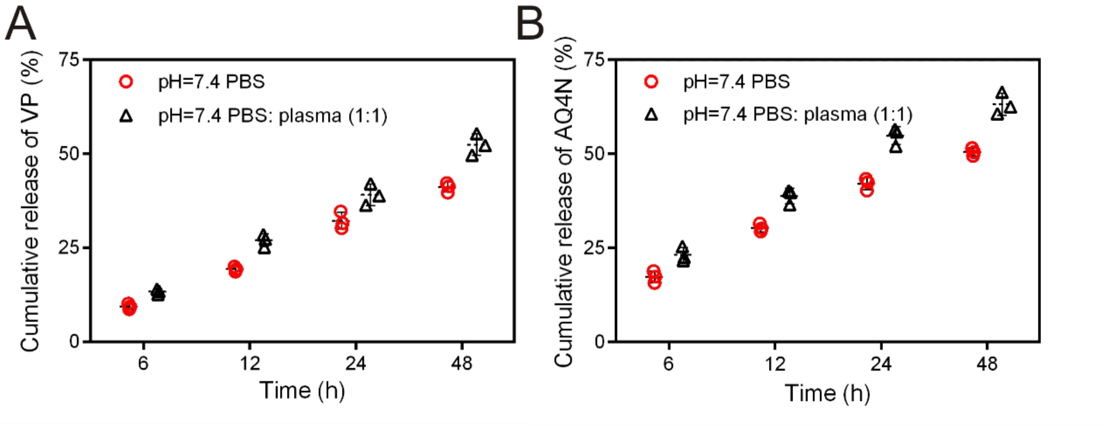


**Fig S3. *In vitro* release of VP (A) and AQ4N (B) from the nanosystem.** The tests were performed in PBS (0.01 M, pH 7.4) at 37 ℃, and also in the mixture of PBS and rat plasma (1:1, v/v), respectively. Briefly, 25 mg of the nanosystem were put in a tube and suspended in 5 mL PBS (0.01 M, pH 7.4) or mixture of PBS and rat plasma (1:1, v/v). The tubes were placed in the gas bath at 37 ℃, and at specific intervals the nanoparticles were centrifuged with Mili-Q membrane filter (4000 rpm, 10 min, 100 kDa). The concentration of VP and AQ4N in the filtration were determined from calibration curves calculated from their characteristic absorbance peaks at 410 nm for VP and 610 nm for AQ4N, respectively. The cumulative release ratios were estimated through dividing the total drug amount by the drug found in the filtration. Data are presented as mean ± s.d. (n=3)

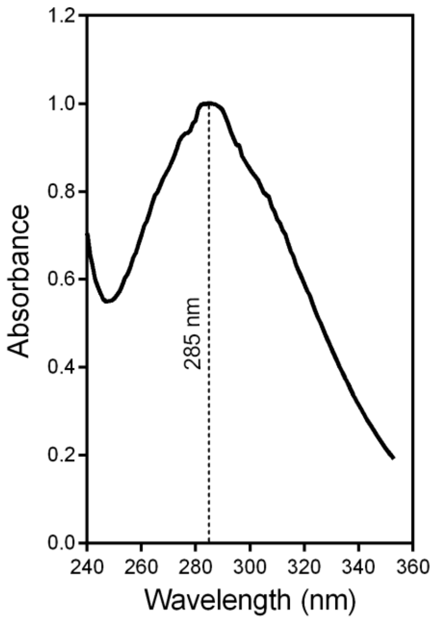


**Figure S4. UV absorption spectrum of PEI/copper (II) complex in water.** The maximal absorption wavelength is at 285 nm.

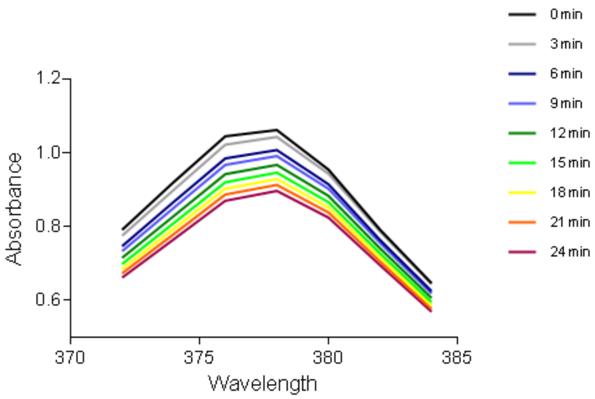


**Figure S5. Photodegradation of ADMA in c(RGDfK)-ppGO/VP (1 µM VP) under 690 nm laser irradiation (30 mW/cm$^2$).** The absorption bands (370 nm~ 385 nm) of ADMA (150 µM) gradually decreased with the irradiation time.

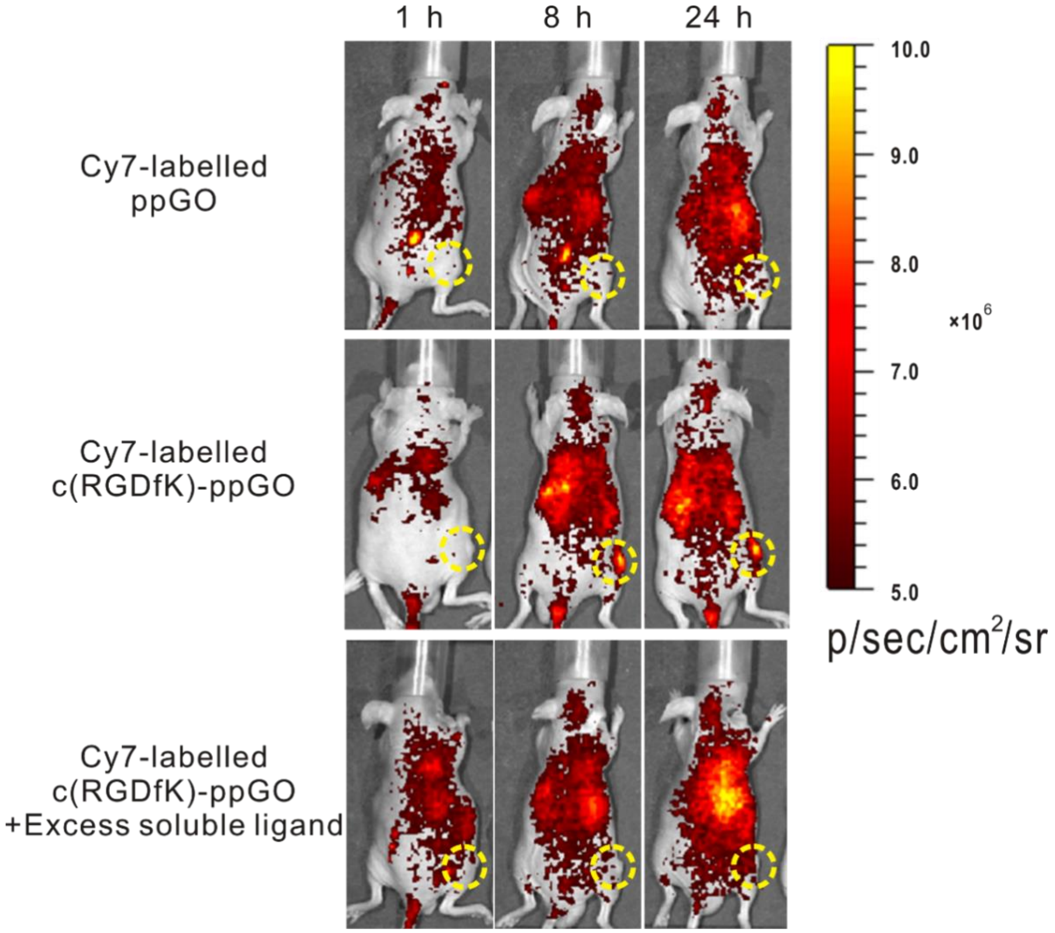


**Figure S6. c(RGDfK) modification facilitated the nanoparticle accumulation in tumors.** Male BALB/c nude mice bearing PC-3 tumor (150 $mm^3$) were given a single intravenous injection of Cy7-labelled ppGO or c(RGDfK)-ppGO, at Cy7 dose of 0.4 mg/kg, or co-injection of Cy7-labelled c(RGDfK)-ppGO and 50 fold molar excess free c(RGDfK) peptides (n=3). Mice with *in vivo* Cy7 fluorescence were imaged at per-determined time after injection using the Xenogen IVIS 200 system, and the representative images at 1 h, 8 h and 24 h were shown. The tumors are indicated in yellow circles.

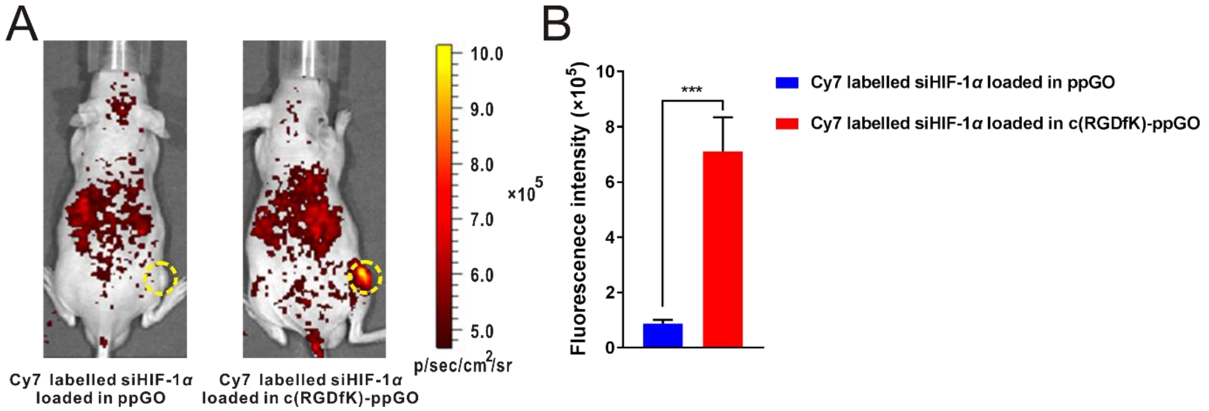


**Figure S7. c(RGDfK) modification facilitated the siHIF-1*α* targeted accumulation in tumors.** (A) The accumulation of Cy7 labelled siHIF-1*α* in tumors at 2 h was examined using the Xenogen IVIS 200 imaging system. The tumors are indicated in yellow circles. (B) Statistical assay of the fluorescence intensity in the tumor regions. Data are presented as mean ± s.d. (n=3) ***$p < 0.001$.

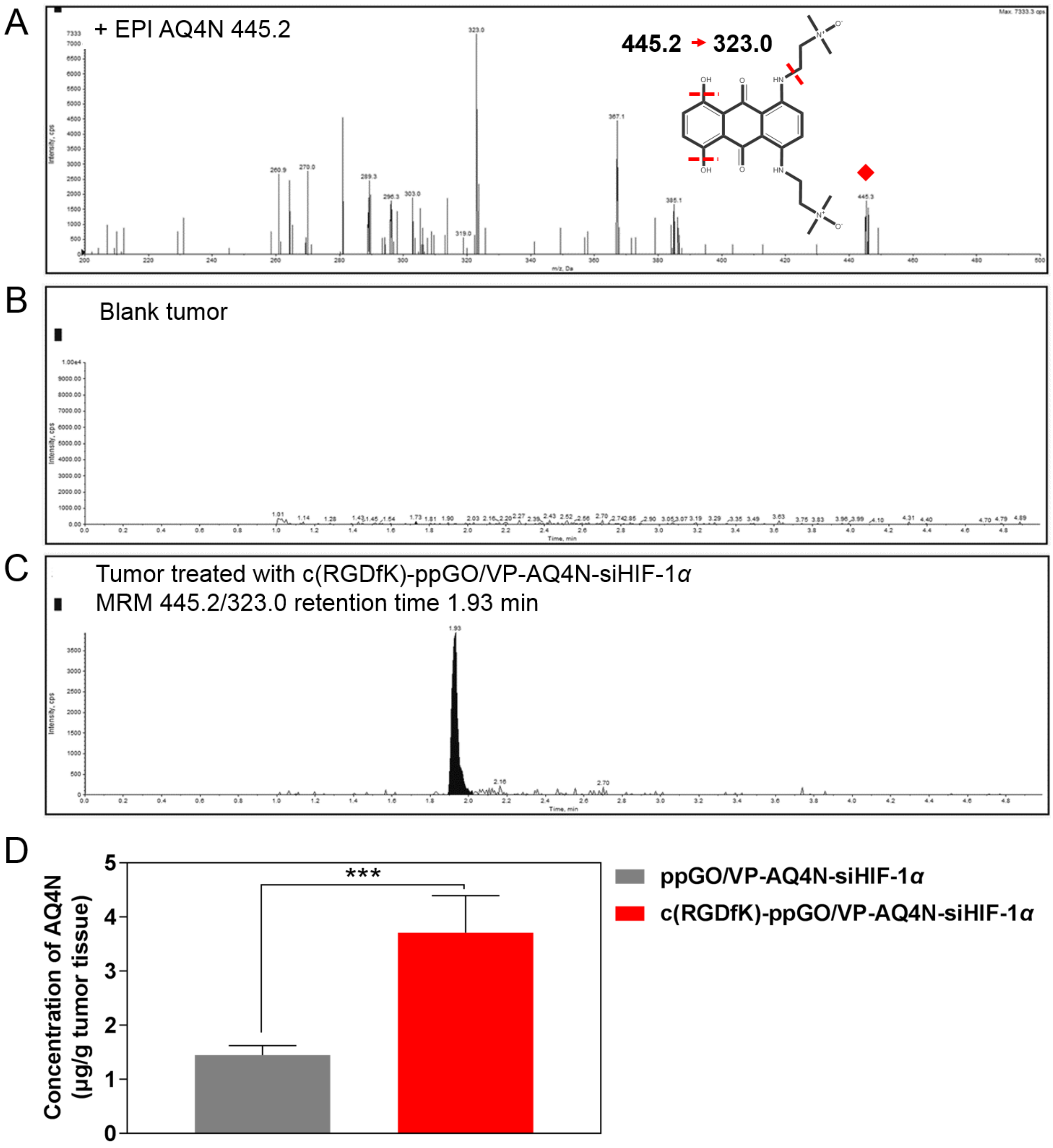


**Figure S8. LC-MS/MS method for the assay of targeted AQ4N distribution in tumors.** (A) Product ion mass spectrum of AQ4N. (B) A blank PC-3 tumor homogenate. (C) A PC-3 tumor homogenate at 2 h after treatment with c(RGDfK)-ppGO/VP-AQ4N-siHIF-1$\alpha$. (D) c(RGDfK) modification facilitated the targeted accumulation of AQ4N in tumors. Data are presented as mean ± s.d. (n=3) ***$p < 0.001$.

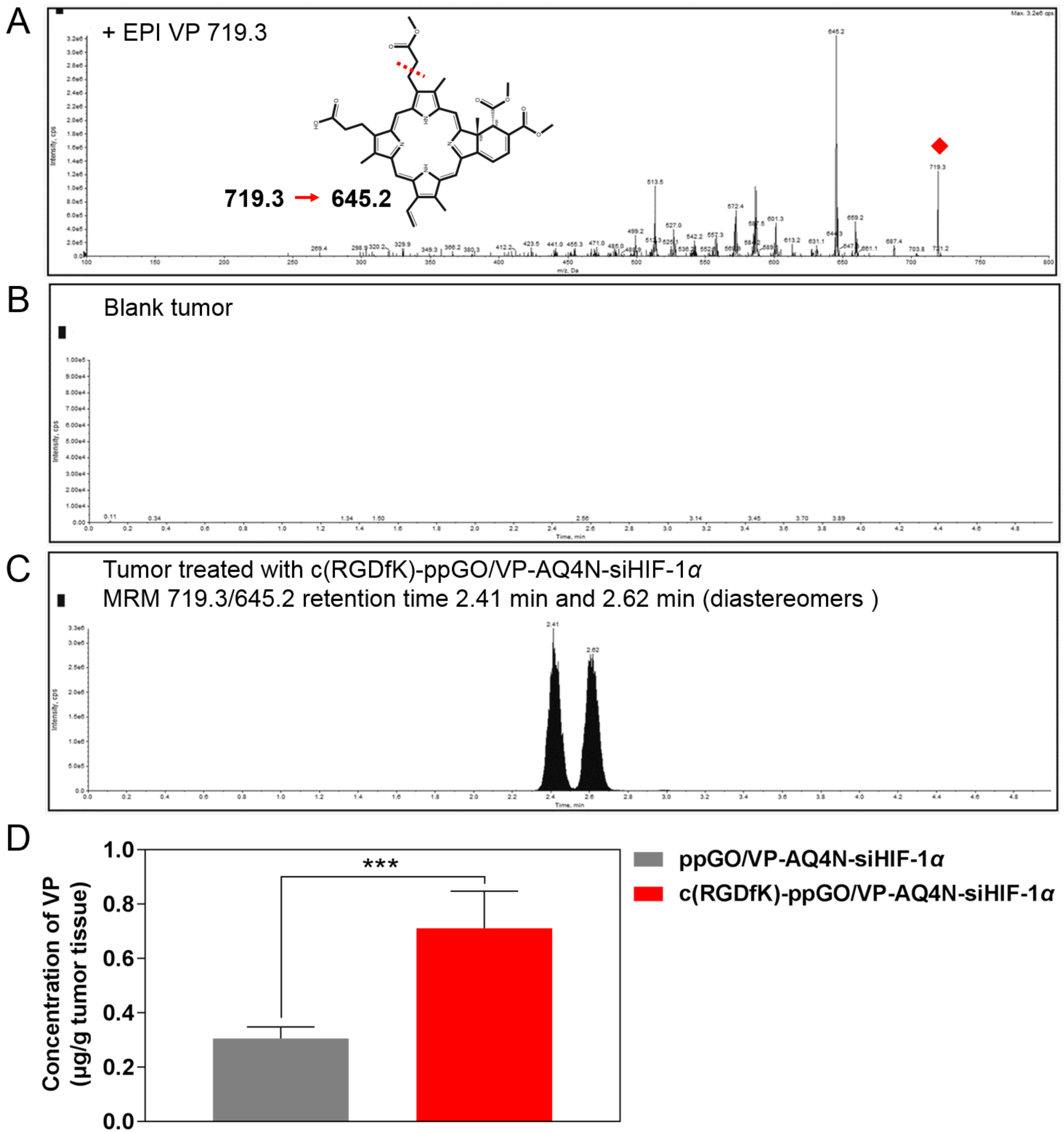


**Figure S9. LC-MS/MS method for the assay of targeted VP distribution in tumors.** (A) Product ion mass spectrum of VP. (B) A blank PC-3 tumor homogenate. (C) A PC-3 tumor homogenate at 2 h after treatment with c(RGDfK)-ppGO/VP-AQ4N-siHIF-1*α*. (D) c(RGDfK) modification facilitated the targeted accumulation of VP in tumors. Data are presented as mean ± s.d. (n=3) ***$p < 0.001$.

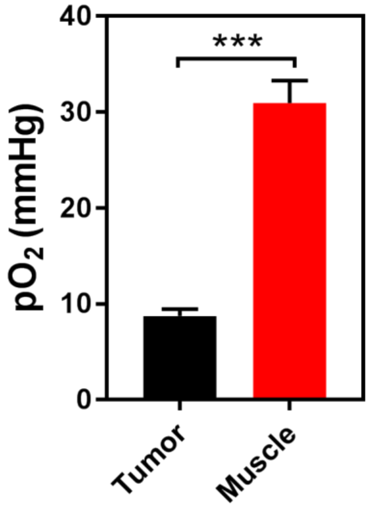


**Figure S10.** The partial oxygen pressure ($pO_2$) of the intact PC-3 tumors at the used size and the muscle of mice hind legs examined using an Oxylite fiber-optic oxygen sensor. The data are presented as mean ± s.d. (n=3) ***$p < 0.001$.

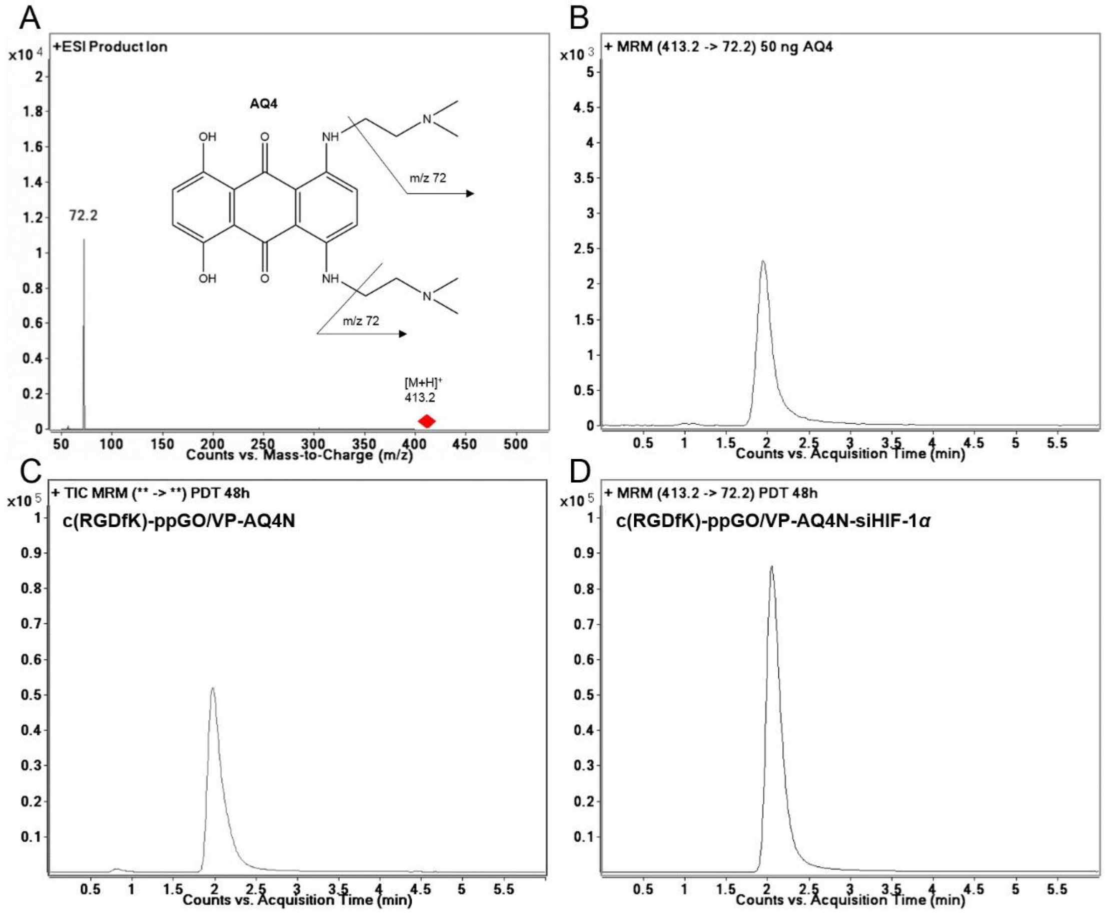


**Figure S11. LC-MS/MS method for AQ4 detection**. Analyses were performed on an Agilent 6410 triple quadrupole mass spectrometer equipped with electrospray ionization (ESI) and an Agilent 1200 HPLC system. A Merck ZIC-HILIC column (2.1 mm × 100 mm, 3.5 μm) was used for analyte separation. Isocratic elution with a mobile phase consisting of acetonitrile and water (60: 40, v/v, the aqueous phase contained 0.1% formic acid and 10 nM ammonium formate, 0.3 mL/min) was used for the separation. (**A**) Product ion mass spectrum of AQ4. (**B**) A blank tumor homogenate spiked with AQ4 (50 ng/mL). (**C**) A PC-3 tumor homogenate at 48 h after treatment with c(RGDfK)-ppGO/VP-AQ4N. (**D**) A PC-3 tumor homogenate at 48 h after treatment with the targeted trimodal nanosystem (c(RGDfK)-ppGO/VP-AQ4N-siHIF-1$\alpha$).

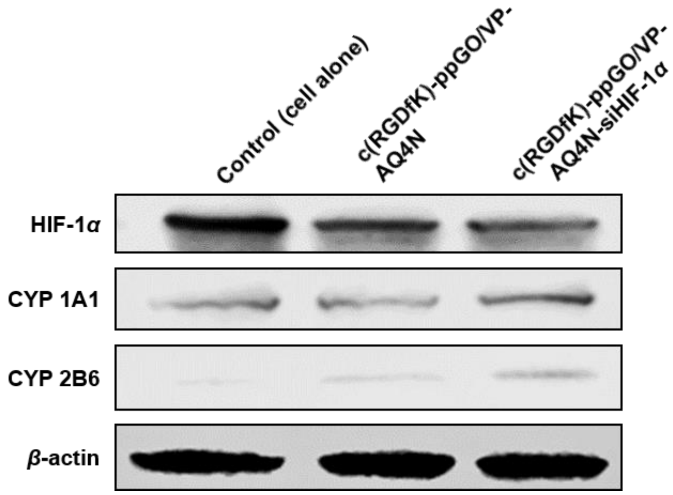


**Figure S12. Effect of siHIF-1*α* on the expression of HIF-1*α* and CYP1A1 and 2B6 in tumors at 24 h after 690 nm irradiation.** At 24 h after irradiation, 3 nude mice from the group of targeted trimodal nanosystem (c(RGDfK)-ppGO/VP-AQ4N-siHIF-1*α*) and c(RGDfK)-ppGO/VP-AQ4N were sacrificed. The PC-3 tumors were excised for western blot assay of the HIF-1*α*, CYP 1A1 and 2B6 proteins. The PC-3 cells alone under hypoxia were used as control.

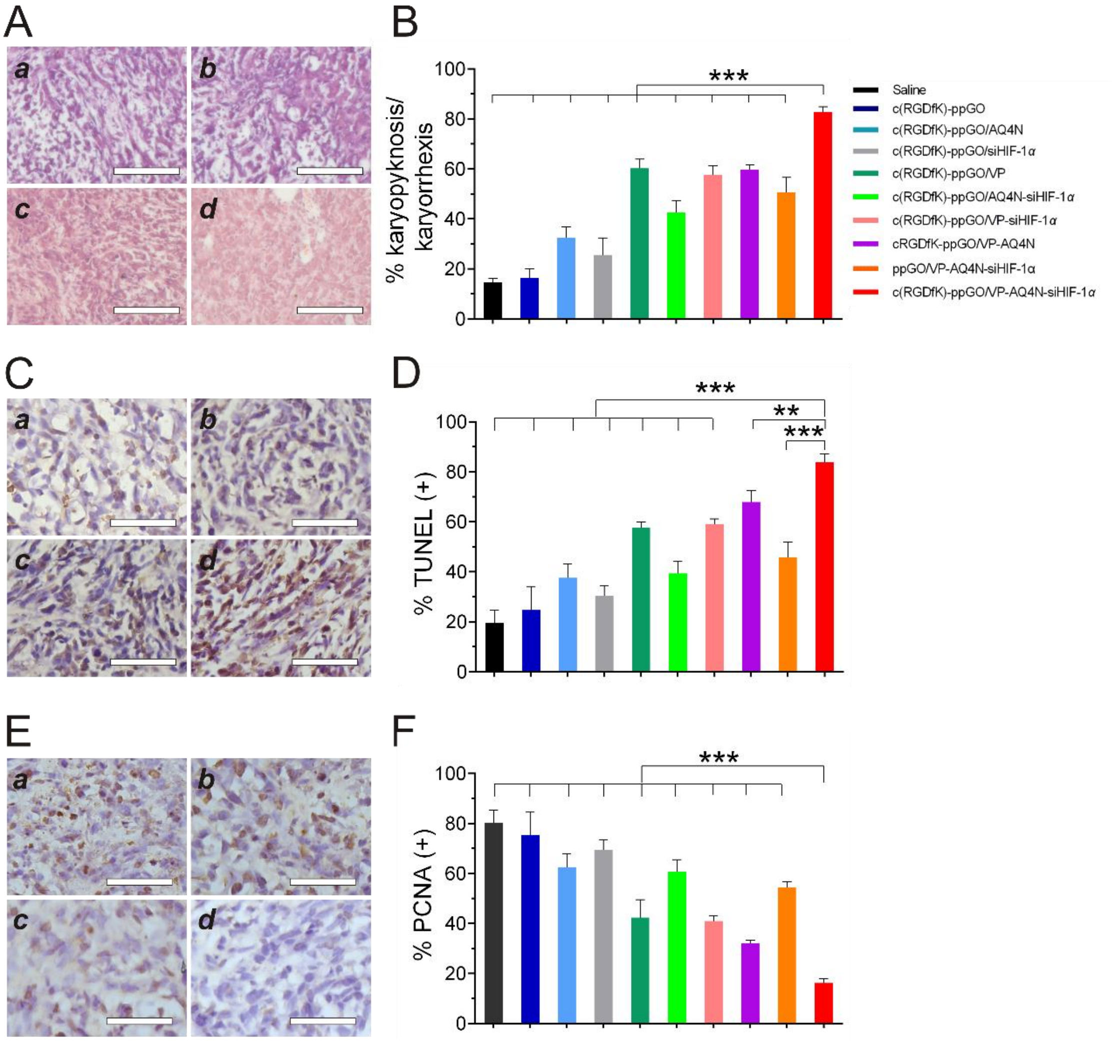


**Figure S13. Histopathological examination of the tumors treated with the targeted trimodal nanosystem.** At 2 h after nanoparticle injection, the tumors were irradiated with laser only once (690 nm, 50 mW/cm$^2$, 20 min). 48 h after irradiation, 3 mice from each group were sacrificed, and the tumors were removed and processed for paraffin sections and histopathological examination. The histopathological photographs were scored blind using the Image-Pro Plus 6.0 software. (**A, B**) H&E staining examinations indicated that more cells were undergoing karyopyknosis and karyorrhexis, the morphological features of apoptotic cells, in the tumors treated with the targeted trimodal nanosystem. Accordingly, the percent of TUNEL positive cells was much higher than those of other groups (**C, D**). In contrast, the PCNA positive cells (**E, F**) were, to the largest extent, inhibited by the targeted trimodal nanosystem. Bar, 50 μm. Italic letter

on the top left corner of each figure refers to Saline (*a*), empty vehicle, i.e. c(RGDfK)-ppGO (*b*), ppGO/VP-AQ4N-siHIF-1$\alpha$ (*c*), and c(RGDfK)-ppGO/VP-AQ4N-siHIF-1$\alpha$ (*d*), respectively. The data are presented as mean ± s.d. (n = 3) $^{**}p < 0.01$, $^{***}p < 0.001$.
